# Fundamental parameters of the developing thymic epithelium in the mouse

**DOI:** 10.1038/s41598-018-29460-0

**Published:** 2018-07-23

**Authors:** Mayumi Hirakawa, Daisuke Nagakubo, Benoît Kanzler, Sergiy Avilov, Brigitte Krauth, Christiane Happe, Jeremy B. Swann, Anja Nusser, Thomas Boehm

**Affiliations:** 1 Department of Developmental Immunology, Max Planck Institute of Immunobiology and Epigenetics, Stübeweg 51, 79108 Freiburg, Germany; 2 Transgenic Mouse Core Facility, Max Planck Institute of Immunobiology and Epigenetics, Stübeweg 51, 79108 Freiburg, Germany; 3 Imaging Facility, Max Planck Institute of Immunobiology and Epigenetics, Stübeweg 51, 79108 Freiburg, Germany; 4 Present Address: Division of Health and Hygienic Sciences, Faculty of Pharmaceutical Sciences, Himeji Dokkyo University, 7-2-1 Kamiohno, Himeji, Hyogo 670-8524 Japan

## Abstract

The numbers of thymic epithelial cells (TECs) and thymocytes steadily increase during embryogenesis. To examine this dynamic, we generated several TEC-specific transgenic mouse lines, which express fluorescent proteins in the nucleus, the cytosol and in the membranes under the control of the *Foxn1* promoter. These tools enabled us to determine TEC numbers in tissue sections by confocal fluorescent microscopy, and in the intact organ by light-sheet microscopy. Compared to histological procedures, flow cytometric analysis of thymic cellularity is shown to underestimate the numbers of TECs by one order of magnitude; using enzymatic digestion of thymic tissue, the loss of cortical TECs (cTECs) is several fold greater than that of medullary TECs (mTECs), although different cTEC subsets appear to be still present in the final preparation. Novel reporter lines driven by *Psmb11* and *Prss16* promoters revealed the trajectory of differentiation of cTEC-like cells, and, owing to the additional facility of conditional cell ablation, allowed us to follow the recovery of such cells after their depletion during embryogenesis. Multiparametric histological analyses indicate that the new transgenic reporter lines not only reveal the unique morphologies of different TEC subsets, but are also conducive to the analysis of the complex cellular interactions in the thymus.

## Introduction

The thymus can be found in all vertebrates and provides a dedicated micro-environment that is essential for T cell development^1^. During development, the thymic epithelium, which represents the major constituent of the thymic microenvironment, emerges from the pharyngeal endoderm as a consequence of reciprocal inductive signals that occur between the endoderm and neural crest-derived mesenchyme and non-crest mesoderm^2^. This stereotypical embryological sequence has been well established for tetrapods, but it is conceivable that the developmental trajectory of the epithelial microenvironment of the thymus also follows the same pattern in fishes, thus rendering the thymus the primary lymphoid organ with the longest verifiable evolutionary history of spatial and functional conservation^3^. The lymphopoietic activity of the thymus^4^ strictly depends on the activity of the *Foxn1* gene^5^ which encodes a transcription factor of the forkhead family expressed in the thymic epithelium^6^. *Foxn1* expression begins at around embryonic day (E) 11.5 in the ventral aspect of the third pharyngeal pouch^7^ and continues in the emerging thymic epithelial cells (TECs) throughout life; however, starting in adolescence, a certain fraction of TECs loses *Foxn1* expression^8–10^. The functional significance of this phenomenon is not known.

A particularly notable feature of the thymus is the fact that the organ grows rapidly during early embryonic and adolescent stages, but then begins to slowly involute^11^, so that in aged animals only residual thymopoietic areas remain in this tissue^12^. It is currently unknown whether thymic involution is a sign of the ageing process – and hence potentially detrimental to immune functions^13^ – or the sign of a beneficial adaptation to the changing requirements of an immune-experienced organism.

The overall size of the thymus is determined by the number of developing thymocytes present in the organ, since the cells of the stromal microenvironment constitute only a minor fraction of all cells in the thymus^14^. The exact numerical relationship between haematopoietic cells and stromal components over time is a matter of long-standing debate; indeed, the results of a recent study suggest that, when using the traditional procedure of enzymatic digestion of thymic tissue and subsequent flow cytometry, the number of thymic epithelial cells is underestimated by at least one order of magnitude^15^. This provocative finding raises a number of questions, particularly with respect to the investigation of cellular heterogeneity within the stromal compartment^16^, some of which are addressed here.

Irrespective of the precise enumeration of cell populations in the thymus, it is a generally accepted fact that the structure of the thymus is highly dynamic, starting out as an alymphoid epithelial rudiment that eventually becomes colonized and remodelled by haematopoietic progenitors. Indeed, reciprocal interactions between haematopoietic cells and stromal cells (a process commonly referred to as lympho-epithelial crosstalk) not only support the maturation of the stromal cells and their differentiation into the two major subtypes – cortical and medullary TECs – but also provide the necessary inductive signals for the development of T lineage cells from undifferentiated precursors^16^. Accordingly, the thymopoietic activity of the thymus (expressed as the ratio of haematopoietic cells to thymic epithelial cells) changes over time, rapidly increasing during the embryonic and perinatal periods, and reaching a peak in adolescence before slowly declining^17^. While this general pattern seems to be true for rodents as well as for humans, it has also been observed that the peak size of the thymus varies between different strains of rats^18^ and mice^19^; a recent study ascribed the striking differences in thymopoietic activity of thymic epithelial cells in two mouse strains to the presence of a co-dominantly inherited genetic determinant(s)^20^.

To date, most quantitative studies focussing on the developmental history of the thymus relied on flow cytometric determination of thymocyte and epithelial cell numbers^21^. In the present paper, we describe a series of new transgenic reporter lines, which were created with a view to addressing some of the outstanding issues of thymus biology. Considering the reticular morphology of TECs, especially of those in the cortical compartment, we were particularly interested in creating reporter lines with nuclear expression of fluorescent proteins in order to be able to precisely enumerate TECs *in situ*. More specifically, we created transgenic lines exhibiting nuclear fluorescence together with expression of a second fluorescent reporter either in the cytoplasm or in cellular membranes. These lines were used to re-evaluate some of the key parameters of the thymic microenvironment. We also created a series of reporter lines driven by *Psmb11* and *Prss16* promoters in order to reveal the trajectory of differentiation of cTEC-like cells. These constructs also allowed us to conditionally ablate such cells; analysis of TECs during early recovery supports the developmental relationship between cTEC-like cells and mature mTECs.

## Results and Discussion

### New transgenic lines with TEC-specific reporter gene expression

In order to direct expression of reporter genes to all thymic epithelial cells (TECs), we exploited the previously characterized *Foxn1* promoter fragment^22^. We generated three new transgenic reporter lines, each co-expressing two fluorescent proteins (Supplementary Fig. 1a). The lines were established by co-injection of two separate expression constructs and screened for co-segregation of the two constructs.

**Figure 1:**
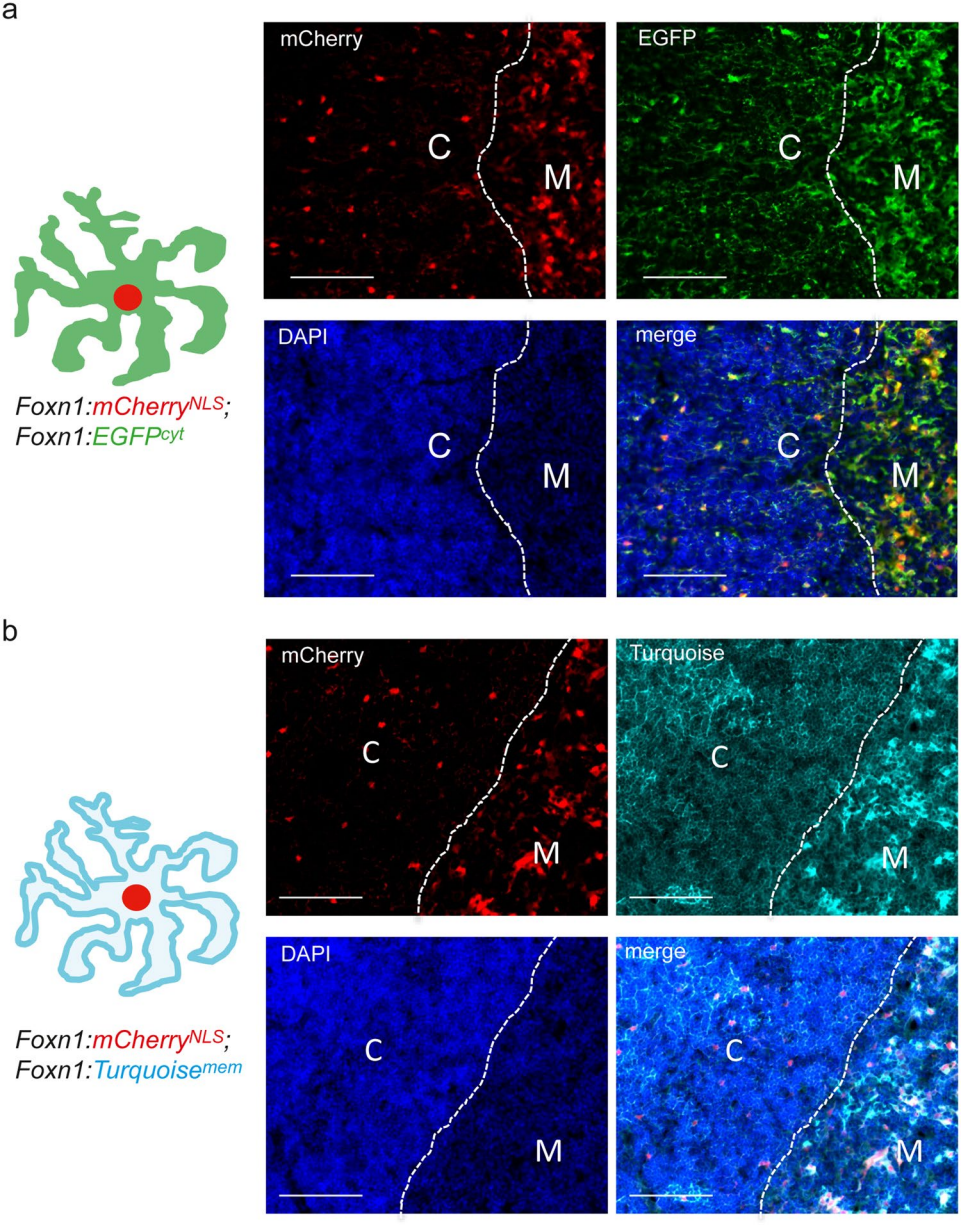
Characterization of new transgenic reporter lines. (**a**) In the *Foxn1*:*mCherry*^*NLS*^; *Foxn1*:*EGFP*^*cyt*^ transgenic thymus of a P10 mouse, nuclei of TECs are red fluorescent, whereas their cytoplasm is green (schematic to the left). The DAPI stain marks nuclei, allowing the distinction of cortex and medulla. The merged image illustrates the markedly different ratios of thymocytes to TECs (thymopoietic index) in the cortex and the medulla. (**b**) In the *Foxn1*:*mCherry*^*NLS*^; *Foxn1*:*Turquoise*^*mem*^ transgenic thymus of a P10 mouse, nuclei of TECs are red fluorescent, whereas their cellular membranes are marked by cyan fluorescence (schematic to the left). Note the presence of an elaborate network of cytoplasmic extensions of TECs in the cortex. Scale bars, 0.1 mm.

In the first line, designated *Foxn1*:*EGFP*; *Foxn1*:*mCherry*^*NLS*^, one construct is equivalent to the previously described *Foxn1*:*EGFP* transgene^23^ directing cytoplasmic expression of EGFP, whereas the other construct (*Foxn1*:*mCherry*^NLS^) directs expression of a red-fluorescent protein^24^ carrying a nuclear localization signal (NLS) and hence accumulates in the nucleus. As expected, in histological sections of transgenic thymi, TECs are marked by bright-red nuclear fluorescence (Fig. 1a). This unique characteristic affords us the possibility of unambiguously determining the number and distribution of Foxn1^+^ TECs in the distinct compartments of the thymus (see below). In addition, the strong cytoplasmic staining emanating from EGFP illustrates the substantial morphological differences between cortical and medullary compartments, marked by substantially different densities of thymocytes, as revealed by DAPI nuclear staining (Fig. 1a).

The second transgenic line, designated *Foxn1*:*mCherry*^*NLS*^; *Foxn1*:*Turquoise*^*mem*^, combines nuclear expression of a red fluorescence protein with expression of a cyan fluorescent protein tagged with a myristoylation signal sequence. As a result, the greater part of the turquoise fluorescent protein becomes localized to cellular membranes^25^, thereby enabling visualization of the intricate reticular network associated with the cytoplasmic extensions of individual TECs *in situ*; this is particularly evident for cTECs (Fig. 1b). This feature is especially useful for the study of intercellular contacts in the thymic microenvironment (see below).

The third transgenic line, designated *Foxn1*:*YFP*^*NLS*^; *Foxn1*:*mCardinal*, was established as alternative to the *Foxn1*:*EGFP*; *Foxn1*:*mCherry*^*NLS*^ strain, and is distinguished by co-expression of a nuclear yellow (YFP^NLS^) protein and a cytoplasmic far red (mCardinal)^26^ fluorescent protein (Supplementary Fig. 1a; see below).

As expected, TECs of all three double-transgenic lines co-express the two fluorescent proteins, as exemplified in the *Foxn1*:*EGFP*; *Foxn1*:*mCherry*^*NLS*^ double-transgenic line for different time points up to postnatal day 10 (P10; Supplementary Fig. 1b,c).

Both our group and others have previously found that a certain fraction of both cortical and medullary TECs becomes *Foxn1*-negative in early adolescence (beginning at about 2 weeks of age); the fraction of *Foxn1*-negative cells (which have a history of *Foxn1* expression, approaches ~40% in the adult thymus^8–10^. This phenomenon is replicated in the transgenic lines described here, since the *Foxn1* gene promoter fragment used for their generation recapitulates the endogenous pattern of *Foxn1* expression)^22^; for instance, as determined by flow cytometry at P10, 93.6 ± 1.5% (mean ± s.e.m.; n = 6) of EpCAM^+^ TECs express the *Foxn1* promoter-driven transgenes; this fraction drops to 81.1 ± 1.9% (mean ± s.e.m.; n = 4) at P28, and to 64.5 ± 2.7% (mean ± s.e.m.; n = 18) at 15 weeks of age and older (Supplementary Fig. 2a). To examine the degree of *Foxn1* down-regulation in cortical and medullary compartments of the epithelium, all TECs with a history of *Foxn1* were marked by Cre-mediated activation of YFP fluorescence in the binary *Foxn1*:*Cre*; *RosaR26LSLYFP* background^27^; the additional presence of the *Foxn1*:*mCardinal* transgene enabled us to monitor acute expression of *Foxn1*. When examined in histological sections of thymi of these triple-transgenic mice at 4 weeks of age, *Foxn1*-expressing TECs are positive for both yellow (an indication of previous *Foxn1* expression) and red (a sign of acute *Foxn1* expression) fluorescence, whereas TECs no longer expressing *Foxn1* appear yellow (data not shown). Flow cytometric analyses of adult *Foxn1*:*Cre*; *RosaR26SLSYFP*; *Foxn1*:*mCardinal* triple-transgenic mice show that loss of *Foxn1* expression occurs in both TEC compartments (Supplementary Fig. 2b), as previously noted^10^; interestingly, the sex of the animals does not appreciably affect the loss of *Foxn1* expression (Supplementary Fig. 2a). Owing to the variable appearance of *Foxn1*-negative cells in the thymic microenvironment of mice older than ~2 weeks, precise numerical assessment of TECs is difficult to achieve using transgenes that report acute *Foxn1* expression.

### The dynamic nature of the thymic epithelial stromal compartment

**Figure 2:**
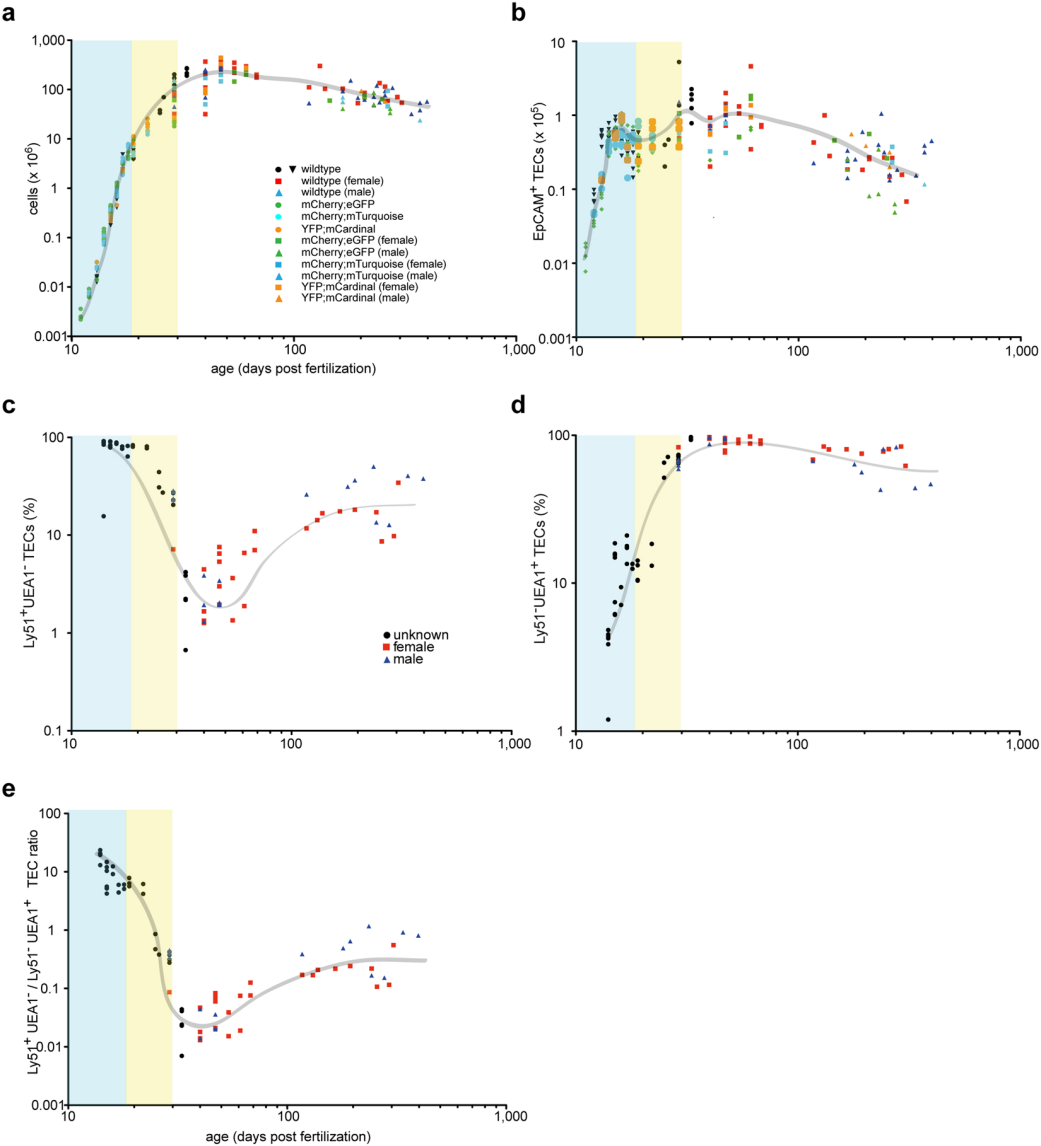
Flow cytometric analysis of thymopoiesis in mice of different ages. (**a**) Total number of cells in the thymus recovered after enzymatic dissociation of tissue. Each symbol represents a single animal; the figure key indicates the genotypes and sex (where known) of each individual; the two wild-type symbols represent mice of two cohorts analysed in different years. The blue shading denotes the embryonic phase, the yellow shading marks the perinatal period until P10. (**b**) Absolute number of EpCAM^+^CD45^−^ thymic epithelial cells determined by flow cytometry. These numbers are similar to those reported in the literature by Jenkinson *et al*.^28^ for E12.5 (~6,300), E15.5 (~45,000), and P28 (~220,000); by Gray *et al*.^17^ for E18.5 (~48,000), P3 (~69,000), P7 (~78,000), P28 (~210,000), indicating the robustness of the classical enzymatic dissociation protocol. (**c**) Fraction of Ly51^+^UEA1^−^ cells among EpCAM^+^ TECs. (**d**) Fraction of Ly51^−^UEA1^+^ cells among EpCAM^+^ TECs. (**e**) Ratio of Ly51^+^UEA1^−^/Ly51^−^UEA1^+^ cells.

Next, we examined well-established parameters of thymopoiesis from embryonic stages to about nine months of age in the newly established transgenic reporter lines. Using the standard procedure of enzymatic dissociation of thymic tissue, followed by flow cytometry, we examined total cellularity, the numbers of CD45^+^ thymocytes and EpCAM^+^ TECs, the latter fraction sub-divided into Ly51^+^ and UEA1^+^ TECs. No differences were found between non-transgenic wild-type mice and transgenic siblings (Fig. 2). The assessment of thymic cell populations by flow cytometry after enzymatic digestion is a procedure reproducible across different laboratories, since previous experiments using the same procedure^17,28^ yielded similar results to the ones presented here. Thymocyte numbers peak in early adolescence and then steadily decline (Fig. 2a); TEC numbers follow a similar general pattern (Fig. 2b). With respect to the components of the epithelial stroma, Ly51^+^ cells exhibit greater fluctuations in numbers (Fig. 2c) than UEA1^+^ cells (Fig. 2d) (see also Supplementary Fig. 2c). A particularly notable feature is the transient reduction of Ly51^+^ TECs in early adolescence; the variable contributions of Ly51^+^ and UEA1^+^ cells to the thymic epithelial microenvironment are most evident when presented as Ly51^+^/UEA1^+^ ratios; the postnatal nadir of the Ly51^+^ compartment is followed by an increase of Ly51^+^ relative to UEA1^+^ cells (Fig. 2e). While these findings confirm the general notion of dynamic age-related changes in the thymic microenvironment (particularly those of Ly51^+^ cells), they also require a more detailed analysis of the developmental trajectory of the thymic epithelium.

### Distribution of TECs in cortical and medullary compartments

**Figure 3:**
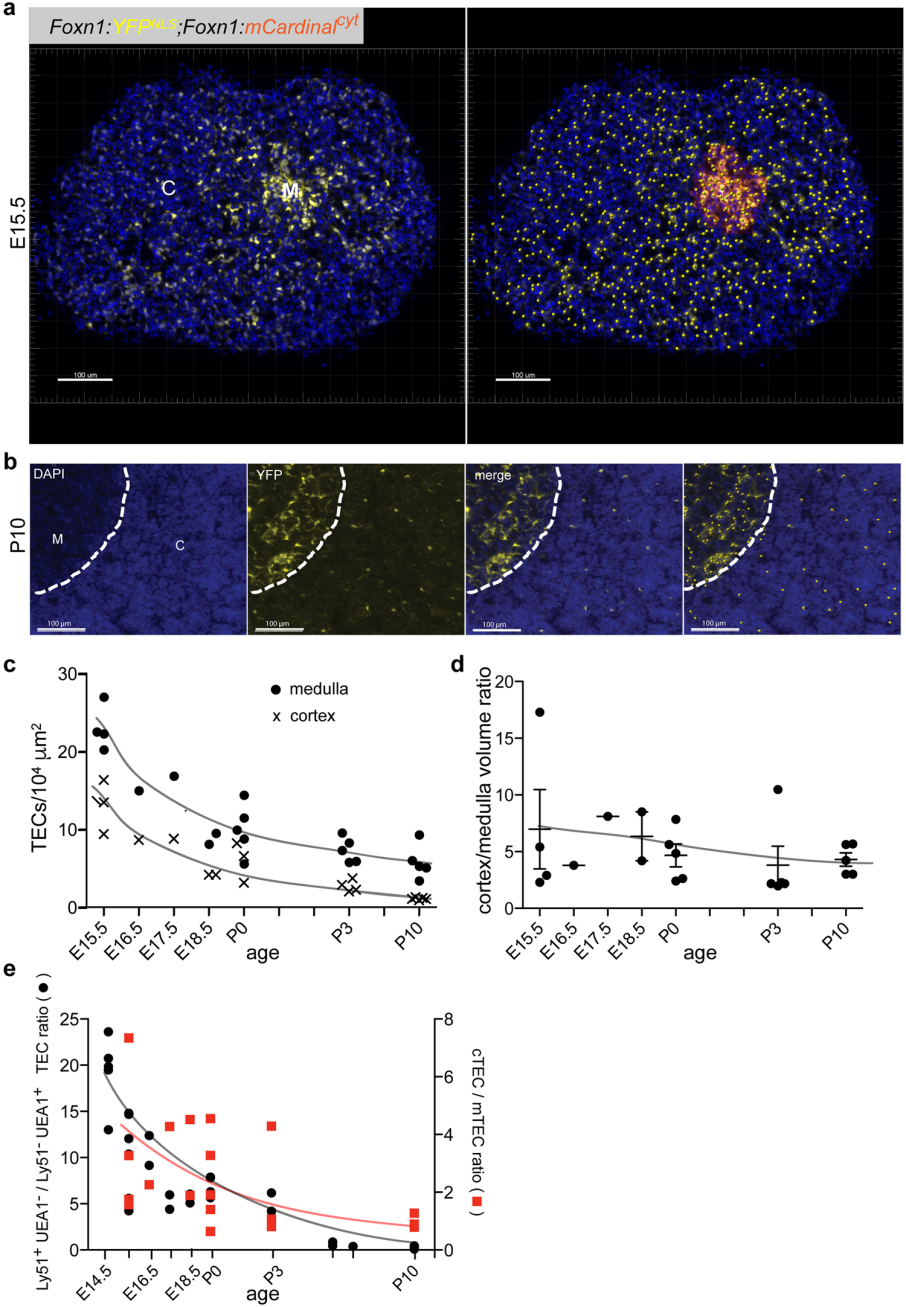
Dynamic changes of TEC densities in the developing thymus. (**a**) Section of a *Foxn1*:*YFP*^*NLS*^; *Foxn1*:*mCardinal*^cyt^ transgenic thymus of a E15.5 mouse; nuclei of TECs are marked with yellow fluorescence, their cytoplasm with red fluorescence. In the left panel, a DAPI stain of nuclei is combined with the yellow nuclear fluorescence; the large cortex and the small medullary region are indicated. In the right panel, DAPI fluorescence is combined with cytoplasmic fluorescence (most clearly recognizable at this magnification for medullary TECs) and nuclei automatically identified by Imaris 8.4.1 software (marked as dots). (**b**) Section of a *Foxn1*:*YFP*^*NLS*^; *Foxn1*:*mCardinal*^cyt^ transgenic thymus of a P10 mouse. Nuclear DAPI and YFP fluorescence signals are shown individually and as a merged image; the right-most panel indicates the software-generated distribution of TEC nuclei. Scale bars, 0.1 mm. (**c**) Density of TEC nuclei in the medullary and cortical regions as a function of age. Each symbol represents a representative section of a single mouse. (**d**) Ratio of medullary and cortical regions as a function of age. Each symbol represents a representative section of a single mouse. In (**c** and **d**), each symbol indicates the value obtained from a single representative section from an individual thymus. (**e**) Comparative analysis of cortical and medullary TECs. Using flow cytometry, the ratio of Ly51^+^UEA1^−^/Ly51^−^UEA1^+^ cells was determined as a proxy for cortical and medullary cells. The cTEC/mTEC ratio was determined from histological sections, taking into account the densities of TEC nuclei and the fractions of cortex and medullary regions in the thymi.

We first examined whether the characterization of TECs by flow cytometry using Ly51 and UEA1 markers after enzymatic dissociation reflects the distribution of TECs in cortical and medullary regions in the intact organ. We focussed on embryonic and early postnatal stages, before appreciable loss of *Foxn1* expression occurs. Exploiting the nuclear fluorescence of TECs in the transgenic lines, we counted cells in tissue sections and calculated the density of TECs in the two compartments. As illustrated in Fig. 3a,b for two different time points (E15.5, and P10), the density of nuclei in the cortical compartment decreases by about one order of magnitude, from ~13 TECs/10^4^ μm^2^ to less than 1 TEC/10^4^ μm^2^; during the same time period, the density of TECs in the medulla decreases about 4-fold, from ~23 TECs/10^4^ μm^2^ to ~5 TECs/10^4^ μm^2^ (Fig. 3c). Despite noticeable regional and inter-individual variability, the dynamic changes in cell densities in cortical and medullary regions of thymi of different ages are accompanied by a gradual reduction of the cortical compartment and a corresponding increase in medullary aspects of the thymus (Fig. 3d). These two developmental phenomena synergize to shift the ratio of cortical to medullary TECs from ~3 at E15.5 to ~1 at P10; this reorganization is reflected in the substantially decreasing Ly51^+^/UEA1^+^ ratios as determined by flow cytometric analyses, falling from ~20 to ~1 during the same time period (Fig. 3e). We conclude that the epithelial microenvironment as assessed by flow cytometry using Ly51 and UEA1 markers reflects, at least in qualitative terms, the dynamic reorganization of the thymic microenvironment. However, assuming that expression of the Ly51 marker accurately reflects the size of the cortical TEC compartment, we note a tendency to underestimate the relative contributions of cTECs to the epithelial microenvironment in the flow cytometric analysis; hence, this phenomenon required further investigation.

### Poor recovery of TECs after enzymatic digestion

**Figure 4:**
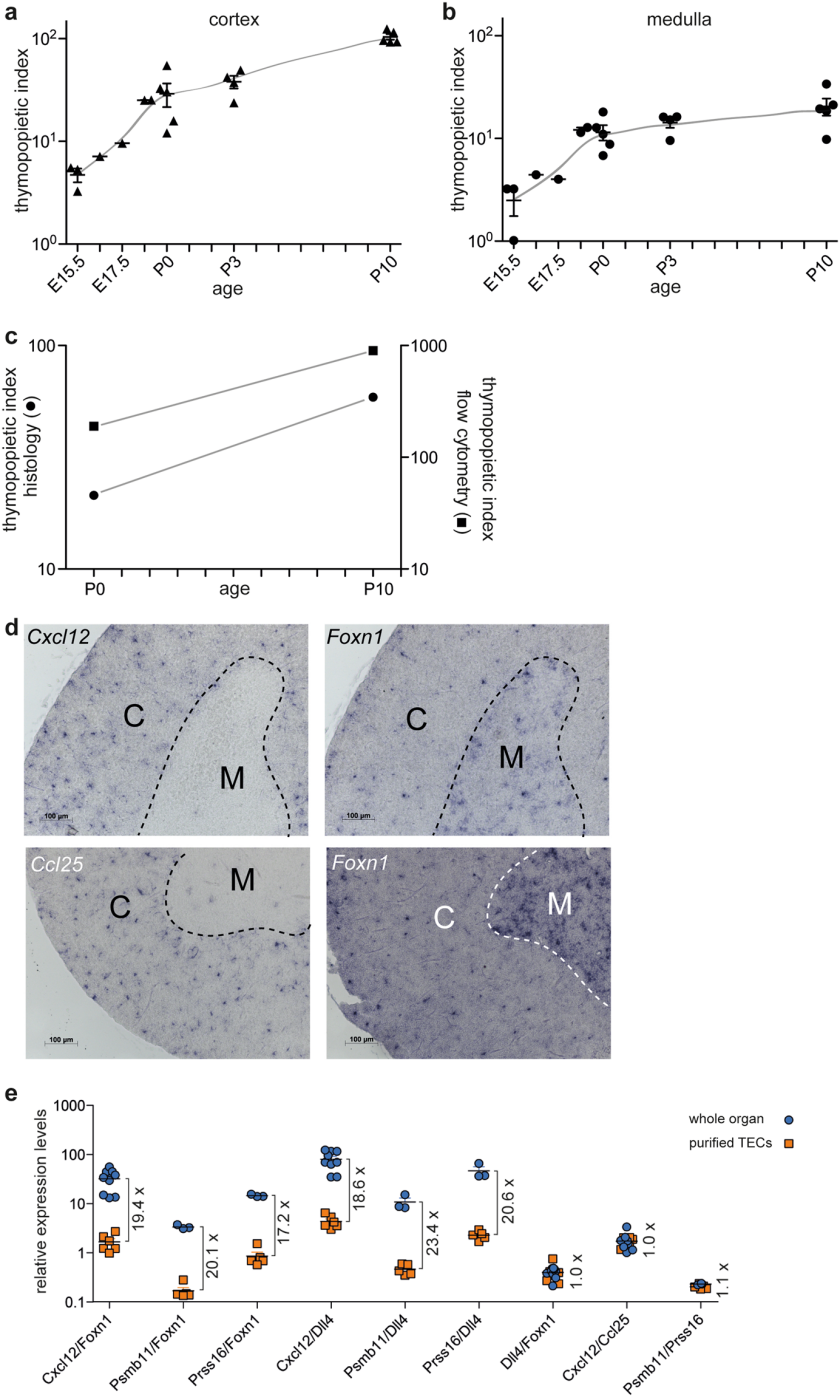
Determinants of thymopoietic activity. Thymopoietic indices of the thymic cortex (**a**) and medulla (**b**) of mice of different ages as determined by histology. (**c**) Comparison of thymopoietic indices determined by histology. (**d**) RNA *in situ* hybridization to localize TECs expressing *Cxcl12*, *Ccl25*, and *Foxn1* in sections of a 4-week-old mouse. The positions of cortex (C) and medulla (M) are indicated. Scale bars, 0.1 mm. (**e**) Expression levels of epithelial-specific genes with a region-specific signature in the thymus using RNA extracted from purified TECs or whole thymi. The fold changes between whole organ and purified TECs are indicated. Since no differences were observed for data of male and female mice (see Supplementary Fig. 4), data were pooled for this analysis.

To this end, we first determined the so-called thymopoietic index (TI) of cortical and medullary regions of the thymus and reconstructed the value of the thymopoietic index for the entire thymus from the proportions of cortical and medullary TECs. Our histological analyses suggest that between E15.5 and P10, the TIs increase for both cortical and medullary regions; the increase is greatest during embryogenesis and then slows down after birth (Fig. 4a,b). Nonetheless, the thymopoietic capacity of the cortex is always substantially higher (about 5-fold at P10) than that of the medulla, the former reaching ~100 at P10. The TI values for the entire thymus as estimated from histological analysis (considering both TEC densities and relative contributions of cortex and medulla) increases by a factor of ~2.7 from P0 to P10, from ~23 to ~61 thymocytes per TEC (Fig. 4c). We then determined the increase in TI values between these two time points by flow cytometry; compared to histological analyses, a somewhat higher increase was observed (~3.8 fold; ~216 to ~810 per TEC) (Fig. 4c). This result indicates that – at least for the perinatal period – both procedures capture the *relative* changes in the thymic microenvironment. However, it is noticeable that the absolute values obtained by histological analyses *in situ* are about 10-fold smaller than those obtained by flow cytometry following enzymatic dissociation. The most parsimonious explanation for the discrepancies observed between histological and flow cytometric measurements is a disproportionate loss of TECs relative to thymocytes during the preparation of TECs. This is most likely attributable to impaired survival owing to the stress imposed by enzymatic digestion, and/or mechanical damage during the subsequent flow cytometric separation of cells. For the 4-week time point, our flow cytometric enumeration yields 1.12 ± 0.12 × 10^5^ TECs (n = 13; mean ± s.e.m.); assuming that we underestimate TEC numbers by at least a factor of 13 (calculated as the ratio of TIs for flow cytometry and histology at P10; Fig. 4c), we arrive at ~1.5 × 10^6^ TECs per thymus. Of note, the TI values measured by the flow cytometric procedure continue to increase during the adolescent period; in adult life, the increase is only modest until about 9 months of age (Supplementary Fig. 3). Unfortunately, owing to variable reduction of *Foxn1* expression in the epithelial compartment past the P10 time point, it proved impossible to reliably compare the values obtained by flow cytometry against those taken from histological sections, although our estimates suggest that the discrepancies between histological and flow cytometric results do not become smaller.

When considering the obvious discrepancies in the outcome of histological and flow cytometric methods, and given the substantially higher TI of cortical versus medullary TECs, we were particularly concerned that the commonly employed procedures used to examine the TEC compartment in enzymatically digested thymic tissue preparations might underestimate the numbers of cTECs in particular, since they are known to tightly associate with immature thymocytes^29–31^. Indeed, the apparent reductions of Ly51^+^/UEA1^+^ ratios as determined by flow cytometry are more pronounced than the changes determined by histological analysis (Fig. 3e). Assuming that the generally accepted gating strategy for Ly51^+^ and UEA1^+^ TECs^21^ accurately reflects the cortical and medullary phenotypes respectively, this observation suggests a preferential loss of cortical TECs relative to mTECs during enzymatic liberation from the organ. Moreover, even for the perinatal period, the relative postnatal increase in the thymopoietic index appears to be higher when measured by flow cytometry than by histological procedures (~3.8 vs. ~2.7; Fig. 4c). To examine this important question further, we compared the expression levels of TEC-specific genes at P28 – a time point at which the thymus has reached adult status – using RNA extracted either from the entire thymus or from TECs purified by preparative flow cytometry. *Cxcl12* was selected as a gene exclusively expressed in the thymic cortex, displaying highest expression levels in the outer cortex; expression of *Ccl25* is more evenly distributed throughout the cortex, and can also be detected in a small number of cells in the medulla (Fig. 4d). Since the expression of both *Cxcl12* and *Ccl25* in TECs is dependent on *Foxn1*^32^, the present analysis is not confounded by the age-related loss of *Foxn1* expression (Supplementary Fig. 2); hence, *Foxn1* was used for comparison, as it is expressed in TECs of both the cortex and the medulla (Fig. 4d). As determined by qPCR, the *Cxcl12/Foxn1* ratios are ~20 times higher for RNA extracted from the entire thymus than for purified TECs; this observation suggests that cTECs are underrepresented in the TEC preparation (Fig. 4e). By contrast, although *Cxcl12* and *Ccl25* exhibit regionally different expression patterns within the cortex, the *Cxcl12/Ccl25* ratios are approximately identical for both sources of RNA, suggesting that TEC preparations faithfully represent the different types of cTECs (Fig. 4e). This observation is supported by subsequent experiments examining the expression levels of *Dll4*, *Psmb11*, and *Prss16*; whereas the former is expressed in both cortical and medullary TECs, the expression of the latter two genes is confined to cTECs (Supplementary Fig. 4). The qPCR analyses confirmed the underrepresentation of cTEC-specific genes in TEC preparations, but revealed no differences for ratios of *Psmb11* and *Prrs16* (Fig. 4e). Collectively, these results suggest that while substantial loss of all types of TECs occurs, this loss is particularly pronounced for cTECs. Therefore, although it appears that all types of epithelial cells will eventually be represented in TEC preparations, caution is required when interpreting the relative contributions of different cell types owing to the systematic biases resulting from the commonly used preparative purification procedures. However, when considered at a qualitative level, our results do not support the possibility that a particular cTEC subset is systematically lost during the commonly used TEC purification procedure.

Next, we addressed the question of the true number of TECs in the thymus at different developmental stages. A recent report has highlighted the discrepancies between histological and flow cytometric assessment of TEC numbers in the adult thymus^15^; given that our results point in a similar direction, we wished to examine this issue using an independent third method. To this end, we measured the number of TECs by light-sheet microscopy after tissue clearing (Supplementary Fig. 5a–c). Recently, light sheet fluorescent microscopy (LSFM)^33^ has been combined with advanced tissue clearing methods^34^ to image the distribution of cells in intact organs. Owing to the considerable technical challenges associated with this type of analysis, here it was conducted for only four time points (E15.5; E16.5; E17.5; and P0). The data indicate an increase in TEC numbers from E15.5 to E17.5, with an attenuated increment until P0 (Supplementary Fig. 5d). However, it was found that the results are very sensitive to the parameters used for the automated determination of cell numbers, most likely owing to the different morphologies of cTECs and mTECs, the histological appearances of which differ substantially, particularly with respect to sizes of the nuclei, as well as cell-to-cell variation of the expression levels of the fluorescent proteins. Notwithstanding these difficulties, it appears that the numbers of TECs extrapolated from histological sections are at least 2-fold higher than those obtained by light-sheet microscopy; by contrast, the TEC numbers as determined by flow cytometry are several-fold lower than those obtained by light-sheet microscopy (Supplementary Fig. 5d). Collectively, the finding that both *in situ* methods result in higher values for total TEC numbers supports the aforementioned conclusion that substantial losses of TECs relative to haematopoietic cells occur during enzymatic digestion of thymic tissue; for instance, at P0, the apparent recovery rate for TECs is less than 10% (Supplementary Fig. 5d). This is compatible with the 10-fold difference in TI values (Fig. 4c) and the results of comparative expression analyses (Fig. 4e).

### Regeneration of the cTEC compartment after subtotal ablation

**Figure 5:**
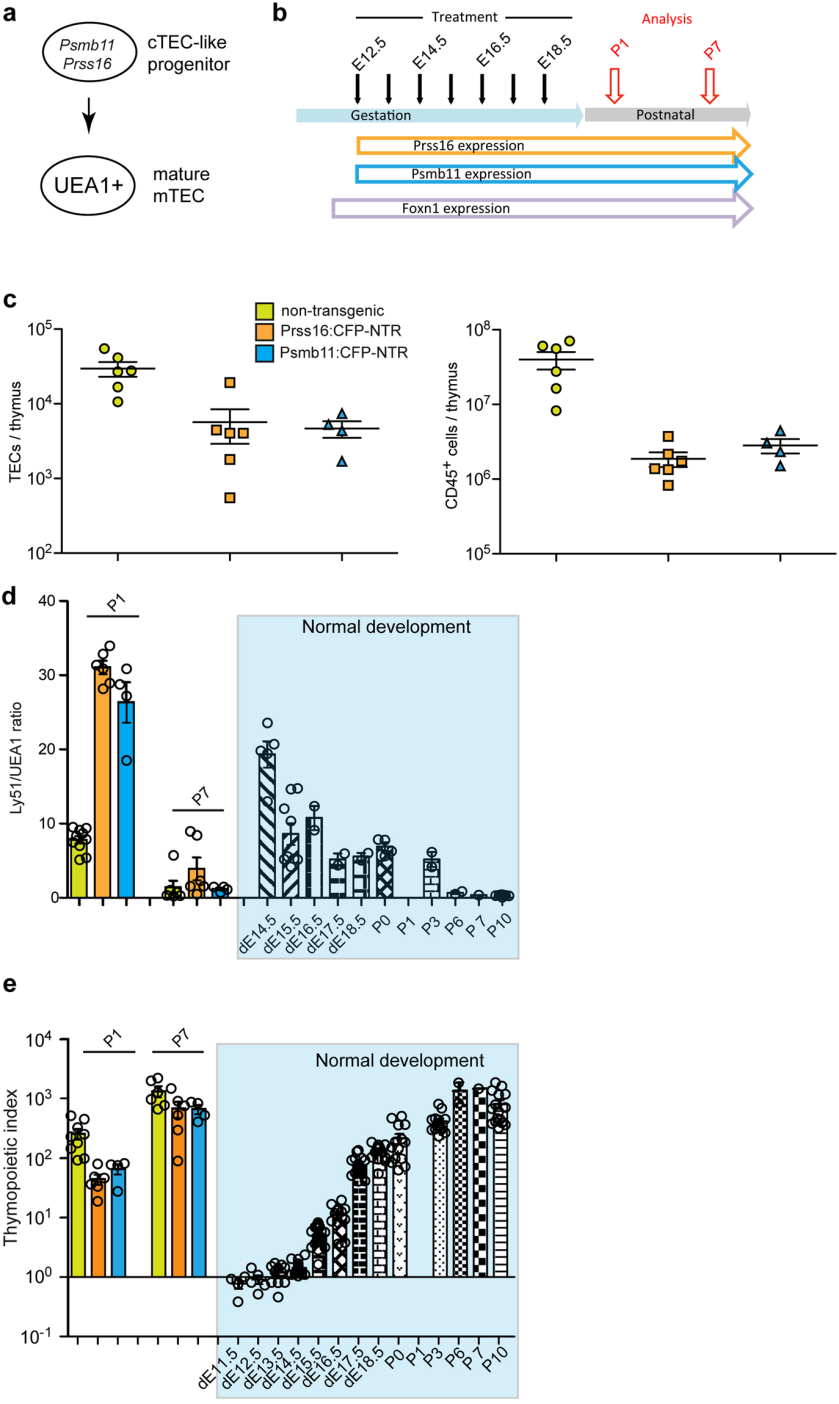
Subtotal ablation of cTEC-like cells and recovery of thymopoietic activity. (**a**) Proposed developmental sequence of cTEC-like cells to mTECs. (**b**) Experimental scheme. Non-transgenic mice pregnant with non-transgenic and transgenic offspring were given daily injections of CB1954. Treatment was terminated at birth (P0). (**c**) Total number of TECs (left panel) and CD45^+^ haematopoietic cells (right panel), as determined by flow cytometry following enzymatic dissociation at P7. (**d**) Emergence of UEA1^+^ TECs during recovery. The emergence of UEA1^+^ cells is accompanied by a reduction of the Ly51^+^/UEA1^+^ ratio. A similar phenomenon occurs during embryonic and perinatal development of wild-type mice (data on the right). (**e**) The increase of thymopoietic activity during the first postnatal week of recovery is equivalent to the increase of the thymopoietic index from E17.5 to P3 of wild-type mice. In (**c**) to (**e**) each symbol represents a single mouse.

Despite the difficulties with precise enumeration, our results indicate that flow cytometric analyses provide a reasonably accurate qualitative representation of the developmental dynamics of the TEC compartment in the thymus. Hence, we continued to examine additional properties of the microenvironment, focussing on the developmental relationship of different TEC subsets. The steady increase in UEA1^+^ TECs (and the concomitant decline of Ly51^+^ cTEC-like TECs) during embryogenesis and the perinatal period (Fig. 2c,d and Supplementary Fig. 2c) supports the notion of a precursor-progeny relationship of cTEC-like cells and mTECs (Fig. 5a), as suggested by several lineage tracing approaches^16^. In order to examine the trajectory and kinetics of TEC differentiation using an independent method, we developed a novel cell ablation model targeting embryonic TECs expressing genes that were previously associated with a cTEC phenotype. We reasoned that if a cTEC-like cell indeed differentiates into UEA1^+^ mTECs, then a reduction of such cells during the embryonic period should lead to the delayed formation of the mTEC compartment postnatally; this should be recognizable by an – at least initially – abnormally high Ly51^+^/UEA1^+^ ratio during the recovery period. Alternatively, if mTECs developed from an independent precursor population, the ratio of UEA1^+^ TECs to Ly51^+^ cTEC-like cells should be altered in favour of UEA1^+^ TECs. Accordingly, we generated two complementary transgenic lines suitable for ablation of cTEC-like cells. Previous lineage tracing experiments suggested that, during the embryonic period, mTECs arise from progenitors expressing *Psmb11*, considered to be also a marker of mature cTECs, encoding the thymus-specific b5t component of the proteasome that is required for efficient selection of CD8^+^ cells^16^. In a complementary approach, we exploited the observation that the *Prss16* gene (encoding the thymus-specific serine protease, TSSP) is expressed in cTECs^35^; lack of *Prss16* impairs positive selection of CD4 single-positive cells, as observed in mice transgenic for an MHC II restricted TCR^36^.

In order to achieve cTEC-specific ablation, the bacterial nitroreductase (NTR, the product of the *nfsB* gene) gene was expressed under the control of *Psmb11* or *Prss16* promoter fragments. This strategy for TEC ablation was successfully used in our previous studies^8,20^, where conditional cell ablation was achieved by provision of the prodrug CB1954, which is converted into a cytotoxic agent by NTR^37,38^. The *Psmb11* promoter fragment contains recognition sites for the Foxn1 transcription factor that are required for the expression of the *Psmb11* gene^39^; whether or not the *Prss16* gene is also under the control of Foxn1 is not known. At E14.5 and later stages of embryonic development, expression of the *Psmb11*:*CFP-NTR* transgene largely overlapped with expression of endogenous Psmb11 protein, as revealed by an anti-Psmb11 antibody; this finding suggests that the promoter fragment used here recapitulates the activity of endogenous regulatory elements of the *Psmb11* gene (Supplementary Fig. 6a). As expected, the *Prss16* promoter fragment used here directs specific expression of the CFP-NTR fusion gene to the cortex, sparing the medullary region; transgene expression largely overlaps with the expression of *Psmb11*, at least during the embryonic stages of development (Supplementary Fig. 6b).

In order to deplete *Psmb11*- or *Prss16*-expressing TECs during embryonic development, non-transgenic pregnant females carrying transgenic and non-transgenic embryos were treated with CB1954. Exposure to CB1954 during the late embryonic period (E12.5 to E18.5) ensures that transgenic and non-transgenic embryos are subjected to exactly the same conditions (Fig. 5b). When litters of treated mice were analysed at P7 (i.e. after one week of recovery), we established that transgenic siblings still exhibited significantly reduced numbers of TECs and thymocytes (Fig. 5c), as we had anticipated, and compatible with the notion that the desired reduction of *Psmb11*- and *Prss16*-expressing TECs was indeed achieved. The Ly51^+^/UEA1^+^ ratio in transgenic mice is strongly biassed towards Ly51^+^ TECs, indicating that, in contrast to the non-transgenic controls, few UEA1^+^ cells are present immediately after the cessation of the cytotoxic treatment (Fig. 5d). After 1 week, the Ly51^+^/UEA1^+^ ratio had fallen, approaching that of wild-type controls (Fig. 5d). When compared with the Ly51^+^/UEA1^+^ ratios observed for the embryonic stages of thymus development, the data suggest that the postnatal recovery period in transgenic mice recapitulates TEC differentiation in a time-delayed fashion (Fig. 5d). The changes observed for the thymopoietic indices reflect a similar pattern; the perinatal increase in TI values is considerably more pronounced in transgenic mice than in their wild-type siblings (Fig. 5e), as expected for a postnatal recapitulation of embryonic development. The composition of thymocyte subsets in the regenerating thymi was similar to that of controls (Supplementary Fig. 7). Collectively, these findings suggest that embryonic TECs expressing *Psmb11* and/or *Prss16* give rise to UEA1^+^ mTECs, in accordance with previous lineage tracing experiments^16^. Since a large fraction of embryonic TECs co-expresses these two genes (Supplementary Fig. 5b), the combination of *Psmb11*:*CFP-NTR* and *Prss16*:*CFP-NTR* transgenes did not have an additive effect on TEC ablation (data not shown). We conclude that the results obtained with the cell ablation model substantiate the progenitor-progeny relationship of cTEC-like and mTEC-like cells during the embryonic period (Fig. 5a)^16^. Moreover, these findings are compatible with the notion of a considerable regenerative potential of the thymic microenvironment, following sub-total ablation; indeed, we had previously observed a similar outcome when cTECs were ablated by injection of diphtheria toxin into newborn mice transgenic for a CCX-CKR1 (atypical non-signalling chemokine receptor CCRL1) promoter-driven human diphtheria toxin receptor (hDTR) expression construct^40^.

### Cellular interactions in the thymic microenvironment

**Figure 6:**
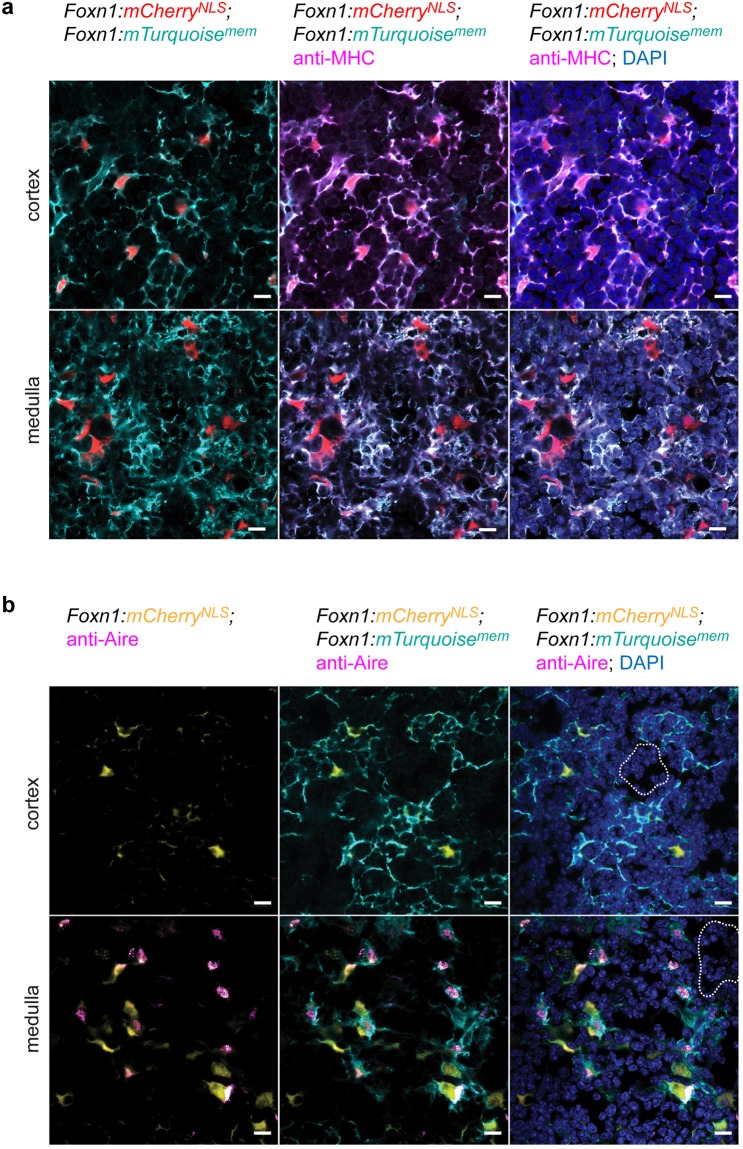
Characteristics of the epithelial microenvironment at P10. (**a**) Co-localization of membrane fluorescence and MHC on the cell surface of TECs in the cortex and the medulla. (**b**) A subset of mTECs but not cTECs expresses Aire protein. Of note, the TEC-specific membrane fluorescence highlights the presence of agglomerations of haematopoietic cells in epithelial-free zones (dotted lines) in both the cortex and the medulla. Scale bars, 0.01 mm.

As demonstrated above, the newly developed transgenic lines have provided us with the opportunity to assess several cytological characteristics and the overall topology of the thymic microenvironment. A particularly useful feature is the robust labelling of the cell surface provided by the *Foxn1*:*mCherry*^*NLS*^; *Foxn1*:*Turquoise*^*mem*^ strain. As expected, the patterns of surface expression of MHCII and the TEC-specific fluorescence of membranes overlap; for instance, at P10, when the morphological differences between cTECs and mTECs are already fully apparent, we observed perfect congruence between cyan membrane-associated fluorescence and anti-MHCII antibody binding at the cell surface (Fig. 6a). The possibility of robustly and uniquely labelling the cell surface of TECs opens up unprecedented opportunities for the analysis of cellular interactions in the thymic microenvironment.

Next, we exploited the possibility of TEC-specific nuclear labelling in the *Foxn1*:*mCherry*^*NLS*^; *Foxn1*:*Turquoise*^*mem*^ strain to examine the distribution of Aire-expressing cells, a previously described subset of mTECs^41^. At P10, the nuclear fluorescence overlaps with anti-Aire antibody staining only in the medullary region, as expected^42^; moreover, the *Foxn1*-associated nuclear fluorescence clearly distinguished Aire-positive from Aire-negative *Foxn1*-positive mTECs, highlighting one intriguing aspect of the functional heterogeneity in this compartment^16,41^.

**Figure 7:**
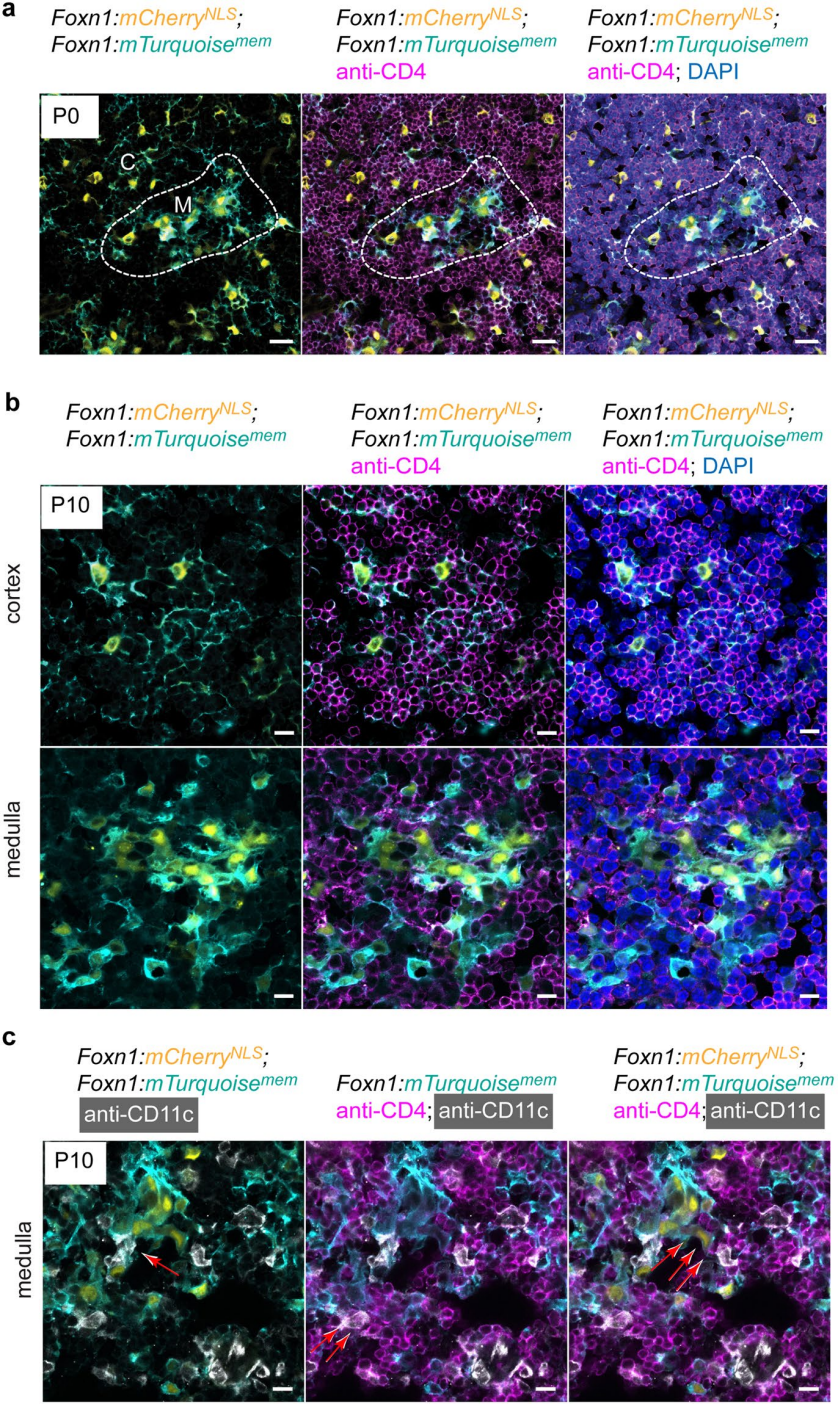
Cellular interactions in the thymic microenvironment. (**a**) Close apposition of CD4+ T cells to cortical (C) and medullary (M) TECs at P0. (**b**) Close apposition of CD4+ T cells to cortical and medullary TECs at P10. Note the substantially higher number of haematopoietic cells per TEC in the cortex. (**c**) Interaction of CD11c^+^ dendritic cells with mCherry/Turquoise-double positive TECs and CD4^+^ thymocytes. Scale bars, 0.01 mm.

To further illustrate the usefulness of the *Foxn1*:*mCherry*^*NLS*^; *Foxn1*:*Turquoise*^*mem*^ strain, we examined the interaction between mTECs, thymocytes, and medullary dendritic cells (DCs). Of particular interest are the modes of interaction of TECs and thymocytes. As described above, at P10, thymocytes outnumber TECs in the cortex by two orders of magnitude. Indeed, the extended reticular network of cTECs ensures that most, if not all, thymocytes are in direct contact with the epithelium, illustrating the previously investigated phenomenon of thymic nurse cells^29–31^ (Fig. 7a,b). Thus, in the future it should be possible to precisely map the characteristics of interactions of these two cell types in unprecedented detail, analogous to the attempts aimed at establishing neural connectivity in different regions of the brain^43^. Moreover, the strong fluorescence of cTEC membranes should prove useful in the development of novel dissociation procedures aimed at liberating cortical thymocytes from their cTEC nursery, without damaging the reticular extensions of the epithelial cells. Interestingly, the morphology and pattern of interaction of mTECs are very different from those of cTECs. The cytoplasmic extensions of mTECs are shorter, and the intercellular distances of mTECs are smaller than in the cortex (Fig. 7b), an arrangement reminiscent of the previously described clonal medullary islets^44^. More generally, the anatomical features of mTECs, particularly the seemingly smaller surface area, explain the lower thymopoietic index in the medullary compartment.

Next, we examined the cellular interactions of dendritic cells (DCs). As previously noted, the vast majority of DCs are found in the medulla. The analysis of the thymic medulla of the *Foxn1*:*mCherry*^*NLS*^; *Foxn1*:*Turquoise*^*mem*^ strain reveals direct interactions between CD11c^+^ DCs and TECs, between CD11c^+^ DCs and thymocytes, and also between thymocytes and TECs (Fig. 7c). This multiplicity of interactions reflects the complex processes underlying the presentation of endogenous antigens during the process of negative selection.

## Conclusion

The new transgenic reporter lines described here have enabled us to reassess a number of fundamental properties of the thymic epithelial microenvironment. Of particular interest was an attempt to determine the number of TECs. For the 4-week time point, our studies indicate that the thymus contains a total of ~1.5 × 10^6^ TECs per thymus. This number compares favourably with the estimate of ~2 × 10^6^ TECs per thymus of a 6-week old mouse provided by Takahama and colleagues using histological techniques and immunostaining for the cTEC-specific b5t component of the immunoproteasome and for Aire as an mTEC-specific marker^15^. Although it seems reasonable to assume that current isolation schemes bring about a greater loss of cTECs than mTECs, the data presented here do not support, but also cannot exclude, the possibility that discrepancies exist among different types of cTECs in the final preparation. Clearly, more detailed studies are required to clarify these issues. Of particular importance would be a transgenic system recording the history of *Foxn1* expression of TECs by nuclear fluorescence. The results of our study also emphasize the need to develop methods which would allow the complete recovery of TECs; this likely requires innovations pertaining to procedures to break the tight association of TECs (particularly of cTECs with their extended reticular processes) with lymphocytes. We anticipate that the use of the reporter strains described here will be a great asset supporting these developments. A second area of innovation should focus on the development of gentle procedures for cell sorting/enrichment since the special morphology of TECs makes them particularly vulnerable to mechanical damage.

## Methods

### Mice

All mice were kept in the animal facility of the Max Planck Institute of Immunobiology and Epigenetics under specific pathogen-free conditions. All animal experiments were performed in accordance with the relevant guidelines and regulations, approved by the review committee of the Max Planck Institute of Immunobiology and Epigenetics and the Regierungspräsidium Freiburg, Germany (licence AZ 35-9185.81/G-12/85).

### Generation of transgenic mice

Expression constructs were injected into FVB/N pronuclei using standard techniques, and the resulting transgenic mice were subsequently backcrossed to a C57BL/6J background. The presence of the transgenes did not result in any health burden.

- Transgenic constructs with *Foxn1* promoter. For the *Foxn1* promoter-driven reporter lines, the pAHB14 plasmid backbone^22^ was used; cDNAs were supplied with NotI-compatible ends and cloned into the unique NotI site of the vector; correct orientation and integrity of the inserts was determined by sequencing. For microinjection, constructs were linearized with SalI. The mTurquoise2^mem^ cDNA^45^ was amplified from plasmid #36209 (Addgene) with primers 5′-GTCGCGGCCGCTTTACTTGTACAGCTCGTCCATGC and 5′-CCA GCGGCCGC CACCATGGTGAGC (NotI restriction enzyme site underlined).
  The mCherry^NLS^ cDNA^46^ was amplified from plasmid #37354 (Addgene) with primers 5′-AAC GCGGCCGCCACCATGACTGCTCC and 5′-GTCGCGGCCGC TTTACTTGTACAGCTCGTCCATGC.
  The YFP^NLS^ cDNA^46^ was amplified from plasmid #37354 (Addgene) with primers 5′-AAC GCGGCCGCCACCATGACTGCTCC and 5′-GTCGCGGCCGC TTTACTTGTACAGCTCGTCCATGC.
  The mCardinal cDNA^26^ was amplified from plasmid #51311 (Addgene) with primers 5′-GAG GCGGCCGC GATCCGATGGTGAGCAAGG and 5′-GAG GCGGCCGC TTACTTGTACAGCTCGTCCATG.
  The *Foxn1*:*EGFP* construct was described previously^23^.
  Transgenic lines were generated by co-injecting two constructs each using standard techniques (Supplementary Fig. 1).
- Transgenic constructs with *Psmb11* promoter. To serve as *Psmb11* promoter, a 2,567 bp genomic fragment of mouse chromosome 14 (nt. 54,627,884–54,625,317) was amplified with primers 5′-GAGGCGGCCGCGTTTAAACTGAGGCTATGGATAAGCAGG and 5′-GCTGGATCCTGAGTGAGAATCGGAAGGAGCAG (BamHI and NotI restriction sites underlined). For microinjection, constructs were linearized with ScaI.
- Transgenic constructs with *Psrss16* promoter. The *Prss16* promoter fragment was generated by amplification of a 2,234 bp region of mouse chromosome 13 (nt. 22,011,974–22,009,740) with primers 5′-GCTGGATCCCTTTTGTCCATCACTGAGAACC and 5′-GAGGCGGCCGCGTTTAAACGAAGACTTAGCTGACAAACCTG (BamHI and NotI restriction sites underlined). These fragments were combined with a CFP-NTR cDNA fragment which was generated by amplification from plasmid tol2_MCS-CFP-NTR^47^ (a kind gift of S. Curado and D. Stainier, University of California, San Francisco) with 5′-CATTCTAGAACTAGTGGATCCACCGGTCGCCACCATGGTGAGCAA and 5′-CATTCTAGATTACACTTCGGTTAAGGTGA (XbaI sites underlined) and cloned into XbaI site of pEGFP-N1 plasmid (Clontech; Genbank accession number U55762), thereby replacing the EGFP cDNA insert.

In addition, the *Prss16* promoter fragment was combined with a cDNA encoding t(andem)d(imer)Tomato which was amplified from plasmid pMYs-IRES-tdTomato^48^; a kind gift of John M. Lindner and Peter Nielsen; this institute) with primers 5′-CATTCTAGAACTAGTGGATCCACCGGTCGCCACCATGGTGAGCAA and 5′-CATTCTAGATTACTTGTACAGCTCGTCCATGCCGTACAGGAAC (XbaI sites underlined) and cloned into the XbaI site of pEGFP-N1 plasmid, as outlined above. For microinjection, constructs were linearized with ScaI.

### Genotyping

The *Foxn1* reporter lines were genotyped using primers 5′-GTCCCTAATCCGATGGCTAGCTC situated in the *Foxn1* promoter fragment (nt 33,509–33,531 in accession number Y12488) and 5′-GTGCAGATGAACTTCAGGGTC, specifically recognizing a sequence common to all cDNA *GFP*-derivative sequences; the sizes of amplicons are as follows: *Foxn1*:*YFP*^*NLS*^, 333 bp; *Foxn1*:*mTurquoise*^*mem*^ and *Foxn1*:*EGFP*^*cyt*^, 410 bp. The *Psmb11*:*CFP-NTR* transgenic mice were genotyped with primers 5′-CTGAGATCAAGCTCTGGTTG (situated in the *Psmb11* promoter sequence) and 5′-GTGCAGATGAACTTCAGGGTC (located in the cDNA sequence); amplicon size is 878 bp. The *Prss16*:*CFP-NTR* transgenic mice were genotyped with primers 5′-CCAGGGTGCCATACATACTA (situated in the *Prss16* promoter sequence) and 5′-GTGCAGATGAACTTCAGGGTC (located in the cDNA sequence); amplicon size is 914 bp. The *Prss16*:*tdTomato* transgenic mice were genotyped with primers 5′-CCAGGGTGCCATACATACTA (situated in the *Prss16* promoter sequence) and 5′- GGCCATGTTGTTGTCCTC (located in the cDNA sequence); amplicon size is 1512 bp.

### TEC ablation

The day of vaginal plug was designated as embryonic day 0.5 (E0.5). 50 mg of CB1954 (C2235; Sigma) was dissolved in 0.5 ml DMSO (100 mg/μl), diluted with PBS to a final concentration of 0.75 mg CB1954/0.1 mL PBS and stored at −20 °C until use. Pregnant female mice were injected i.p. each day with 0.75 mg of CB1954 per day from E12.5 until E18.5. Postnatal mouse thymi were analysed at P1 or P7, respectively. The use of these transgenes for postnatal ablation of cTECs proved difficult owing to transgene-specific toxicity in the skin (*Prss16*:*CFP-NTR*) or the need for large doses of prodrug with apparent non-specific toxicity (*Psmb11*:*CFP-NTR*).

### TEC analysis by flow cytometry

The day of vaginal plug was designated as embryonic day 0.5 (E0.5). Thymic epithelial cells were isolated as follows: To generate single cell suspensions for TEC staining, thymi were finely minced with scissors, and then digested with a cocktail of collagenase type 4 (200 µg/mL), neutral protease (200 µg/mL) and DNaseI (500 ng/mL) in RPMI 1640 + 2% FCS for up to 90 minutes at 37 °C with gentle agitation. Digestion was routinely carried out in a final volume of 1 ml per thymic lobe. Care was taken to keep the small tissue fragments afloat during the early phases of the digestion process to facilitate penetration of enzymes into the tissue; in some instances, the digestion was carried out in two sequential steps, removing the liberated cells from the supernatant after half the incubation time and adding fresh digestion buffer. Following digestion, EDTA was added to a final concentration of 2 mM, which facilitates the disaggregation of any remaining small cell clusters of epithelial cells, presumably owing to the disruption of E-cadherin complexes. In this way, the entire thymic tissue dissociated into a single cell suspension, avoiding isolation/quantification artefacts associated with possible strain-dependent differences in the composition of the thymic microenvironment. Cells were then washed with RPMI 1640 + 2% FCS, and re-suspended in PBS supplemented with 0.5% BSA for staining. Cell surface staining (anti-CD45 [30-F11], conjugated with PE Cy7 [Bio-Legend]; anti-EpCAM [G8.8], conjugated with APC [Bio-Legend]; anti-Ly51 [6C3], conjugated with PE [eBioscience]; UEA1, conjugated with FITC [Vector Biosciences]) was performed at 4 °C in PBS supplemented with 0.5% BSA and 0.02% NaN_3_. Thymic epithelial cells have the surface phenotype CD45^−^/EpCAM^+^; thymocytes are CD45^+^/EpCAM^−^. Note that the enzymatic cocktail required to liberate thymic epithelial cells destroys the extracellular domains of CD4 and CD8 surface markers (but not that of the CD45 molecule); hence, when analysis of thymocyte subsets is required, thymocyte suspensions must be prepared in parallel by mechanical liberation, best achieved by gently pressing thymic lobes through 40 µm sieves. For the calculation of the thymopoietic index, the number of CD45^+^/EpCAM^−^ cells was divided by the number of CD45^−^/EpCAM^+^ cells; the index is equivalent to the inverse of the fraction of CD45^−^/EpCAM^+^ cells in the flow cytometric profile (for instance, a fraction of 0.1% of CD45^−^/EpCAM^+^ cells in a preparation is equivalent to a thymopoietic index of 1,000).

For flow cytometric evaluation of thymocyte subsets, cells were stained with anti-CD4 (clone RM4–5, conjugated with APC [eBioscience]) and anti-CD8α (clone 53–6.7, conjugated with PeCy7 [eBioscience]).

### Passive clearing technique (PACT)

Passive clearing of thymic lobes was carried out as described previously^34^. The refractive index matching solution (RIMS) containing 70% (w/v) sorbitol in 1xPBS (final refractive index ~1.43) was used as clearing mounting medium^49^ instead of the RIMS solution^34^.

### Immunohistochemistry

Thymi were fixed in PBS-4% paraformaldehyde for 2 h at 4 °C and washed with PBS and incubated overnight in PBS containing 20% sucrose. Thymi were embedded in OCT, frozen on dry ice and cut on a Leica C3050S cryostat (section thickness 8 µm) at −20 °C. For each thymus, two to three representative sections were imaged; the volumes of cortex and medulla were calculated from averaged cortical and medullary areas considering the total volume of the thymus, as determined by volume displacement (E16.5, 2.1 ± 0.1 μl [mean ± s.e.m.; n = 7]; P0, 10.6 ± 0.5 μl [mean ± s.e.m.; n = 4]; P10, 55.5 ± 3.5 μl [mean ± s.e.m.; n = 2]; P28, 130.3 ± 4.8 μl [mean ± s.e.m.; n = 8]; E16.5, 2.1 ± 0.1 [mean ± s.e.m.; n = 7];). Histological sections were blocked with PBS containing 10% normal donkey or goat serum for 30 min at RT and subsequently incubated with a mix of primary antibodies in PBS supplemented with 0.5% BSA, 0.2% Tween20, 10% donkey or goat serum. Sections were incubated in a humid chamber for 2 h at RT or occasionally overnight at 4 °C. After washing with PBS, sections were incubated with secondary antibodies in PBS supplemented with 0.5% BSA 0.2%, Tween20, 10% normal donkey or goat serum for 1 h at RT and then washed with PBS. Sections were mounted in Fluoromount-G with or without DAPI. The following were used as primary antibodies: anti-CD4 conjugated with APC (clone RM4-5) (eBioscience); anti-CD11c conjugated with Alexa Fluor 647(N418) (eBioscience); anti-Aire (5H12) anti-mouse Aire^50^ (kindly provided by Dr. Hamish Scott); anti-MHC Class II (I-A/I-E) conjugated with Alexa Fluor 700 (M5/114.15.2) (BioLegend); sheep polyclonal anti-jellyfish GFP (Bio Rad); rabbit polyclonal anti-mouse beta5t (MBL). Secondary antibodies were purchased from Jackson Immunoresearch; donkey anti-rat IgG conjugated with Cy3; donkey anti-rat IgG conjugated with Cy5; goat anti-rat IgG conjugated with Alexa Fluor 647; donkey anti-sheep IgG conjugated with Alexa Fluor 488.

### Microscopy and image analysis

Datasets of 3D images of cleared organs were acquired on Z1 Light Sheet microscope (Zeiss). Thymic tissues were transferred into 2% liquid low melting agarose in a 1 ml syringe; upon solidification of agarose, the syringe was fixed in the sample holder of the microscope, and the agarose cylinder containing the embedded thymus pushed out into the path of the light beam. Images of mCherry fluorescence were acquired with 561 nm excitation, alternating dual side illumination, standard beam-splitter and filter combinations, and the detection objective Clr Plan-Neofluar 20x/1.0 Corr (a dipping objective designed for clearing mounting media with a refractive index of 1.45). For image recording, the PCO. Edge cooled sCMOS camera (PCO, Germany) was used; spatial sampling was adjusted by additional zoom optics to 0.229 µm/pixel in *xy* and 0.589 µm/pixel in *z* dimensions, respectively. When thymi did not fit the single field of view, “tiled” images were acquired, followed by stitching with Vision 4D software (Arivis). Automatic counting of cells in light sheet datasets possibly underestimates the numbers due to non-optimal segmentation, *i*.*e*., failure to separate cells that are in close apposition in z direction, especially cells containing mCherry fluorescence also in the cytoplasm; by contrast, overestimation of cell numbers appears unlikely, since careful examination of the light sheet images did not reveal any false-positive bright spots which could be mistaken by the software for nuclei. Immunohistochemical images were acquired on AxioImager.Z1 Apotome2 and LSM780 microscopes (both Carl Zeiss). For the Apotome2 microscope, standard filter sets for the above-mentioned dyes were used and images acquired through a Plan-Apochromat 20x/0.75 objective with an Axiocam MR R3 CCD camera, yielding a final spatial sampling of 0.645 µm/pixel. Where necessary, tile images were acquired and stitched. On the LSM780 microscope, multi-channel images were acquired sequentially, with the following laser excitation lines: 405 nm for DAPI and cyan; 488 nm for Alexa Fluor 4888 and EGFP; 561 nm, for Cy3, Alexa555; and 633 nm for Cy5, with standard emission ranges for these dyes, using a Plan-Apochromat 63x/1.4 oil objective, with spatial sampling of 0.07 µm/pixel. To count cells expressing mCherry and YFP fluorescent proteins in tissue sections and cleared total thymi, we used the Spots module of the Imaris 8.4.1 software (Bitplane). Spot detection parameters were manually optimized to ensure that “positive” cells are detected as single spots, while minimizing false-positive detection. To measure areas of cortical and medullary regions in the images of tissue slices, the Surfaces module of the Imaris 8.4.1 software was used.

### mRNA *in situ* Hybridization

Thymi were fixed with modified Davidson’s fluid and dehydrated with Li-EtOH and EtOH in a stepwise fashion and finally embedded in paraffin; RNA *in situ* hybridization (ISH) on paraffin sections was performed using DIG-labelled probes^32^. The following probes were used: *Ccl25* (nt 119-553 in Genbank accession number NM_009138); *Cxcl12* (nt 64-479 in Genbank accession number NM_ 021704); *Foxn1* (nt 2181-3584 in Genbank accession number XM_006532266.3); *Dll4* (nt 2271-3421 in Genbank accession number BC049130); *Prss16* (nt: 297-700 in Genbank accession number NM_019429.2); *Psmb11* (nt 394-862 in Genbank accession number AB299436.1).

### qRT-PCR

RNA was isolated from total thymus tissue or from purified thymic epithelial cells (CD45^−^EpCAM^+^) as described^51^. cDNA was prepared using random hexamers and subjected to qPCR (TaqMan) using the following primers: *Foxn1*, 5′-CTCTTCCCAAAGCCCATCTA and 5′-AGGCTTCCGGTCTTACTGTTC; *Ccl25*, 5′-GAGTGCCACCCTAGGTCATC and 5′-CCAGCTGGTGCTTACTCTGA; *Cxcl12*, 5′-CCAAACTGTGCCCTTCAGAT and 5′-ATTTCGGGTCAATGCACACT; *Prss16*; 5′-GCCACTGGATCCCTTCAAT and 5′-CTGTGTTGATCATTCACCCAGT; *Psmb11*, 5′-CTGTGGCTGGGACCACTC and 5′-ATCTCCCTGCAGACAAGTGC; *Dll4*, 5′-AGGTGCCACTTCGGTTACAC and 5′-GGGAGAGCAAATGGCTGATA; *Epcam*, 5′-AGAATACTGTCATTTGCTCCAAACT and 5′-GTTCTGGATCGCCCCTTC. In the PCR reaction, primers were used at a concentration of 10 pmol/µl. Expression levels were normalized to *Epcam*.

### Statistical analysis

t-tests (two-tailed) were used to determine the significance levels of the differences between the means of two independent samples, considering equal or unequal variances as determined by the F-test. For multiple tests, the Bonferroni correction was applied.

## Electronic supplementary material

Supplementary Information
