# Supplementary material for: Fundamental parameters of the developing thymic epithelium in the mouse
Source: Sci Rep. 2018 Jul 23;8:11095. doi: 10.1038/s41598-018-29460-0 (PMC6056470; doi:10.1038/s41598-018-29460-0)
Supplement: Supplementary file 1 — Supplementary Information [file 41598_2018_29460_MOESM1_ESM.pdf]

Supplementary information

Fundamental parameters of the developing thymic epithelium in the mouse

Mayumi Hirakawa, Daisuke Nagakubo, Benoît Kanzler, Sergiy Avilov, Brigitte Krauth, Christiane Happe, Jeremy B. Swann, Anja Nusser, & Thomas Boehm

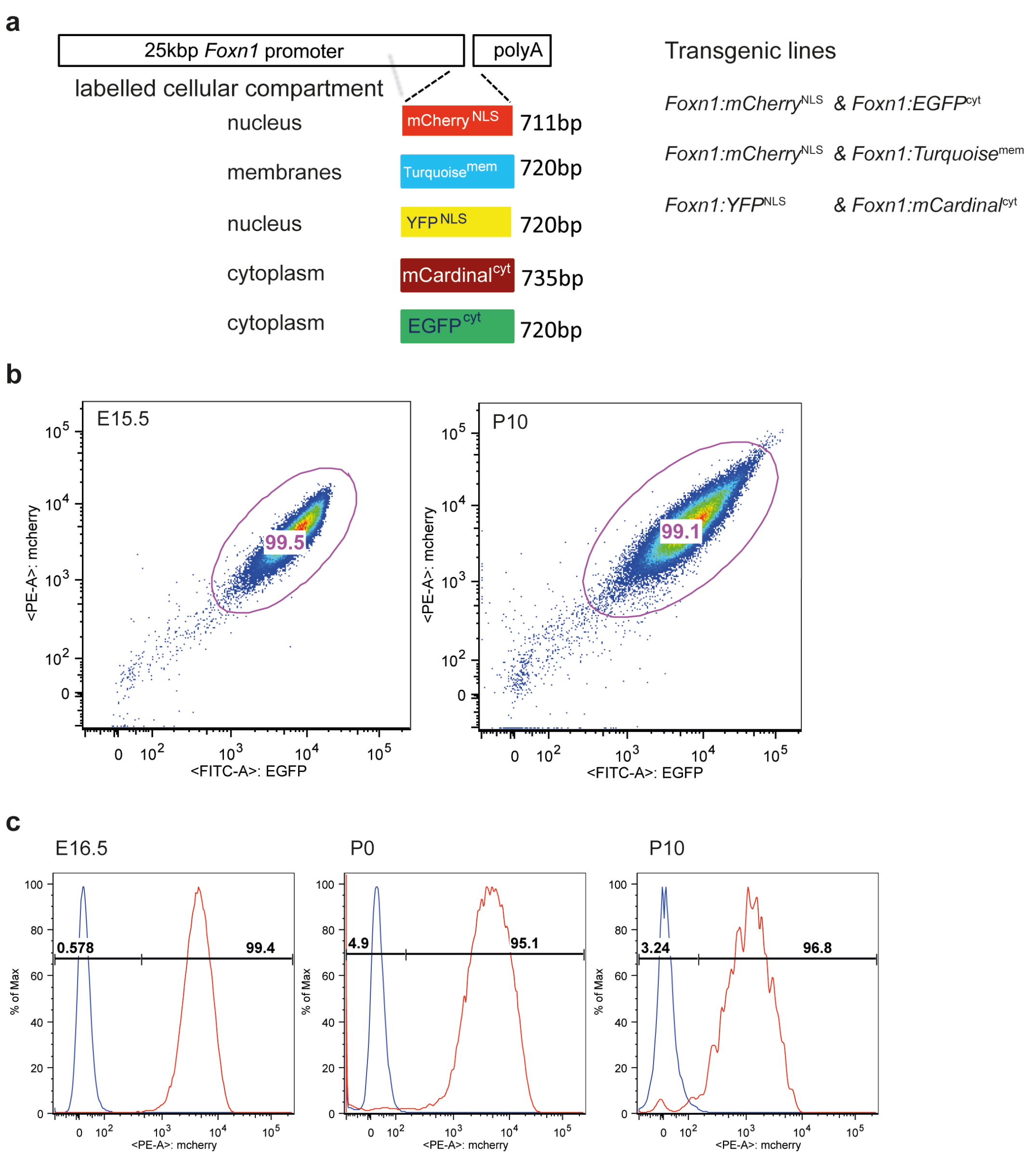

Supplementary Figure 1. Characterization of novel transgenic reporter lines. (**a**) Schematic outline of transgenic constructs, consisting of a ~25kb Foxn1 promoter fragment, cDNA fragments encoding different fluorescent proteins, and poly(A) addition signal (see Methods). The three co-injected (and subsequently co-integrated) construct combinations are indicated. Although not exploited here, the transgenic line expressing the mCardinal protein should enable non-invasive monitoring of the TEC compartment[26]. (**b**) Co-expression of different fluorescent proteins in TECs of the *Foxn1:mCherry*^{NLS}*;Foxn1:EGFP*^{cyt} transgenic line at two time points; cells liberated from enzymatically dissociated thymi were stained and the depicted plots are gated on EpCAM^{+};CD45^{-} cells. (**c**) All EpCAM^{+};CD45^{-} cells of *Foxn1:mCherry*^{NLS}*;Foxn1:EGFP*^{cyt} transgenic thymi express the fluorescent proteins

up to at least P10. Blue histograms are from non-transgenic, red histograms are from transgenic mice.

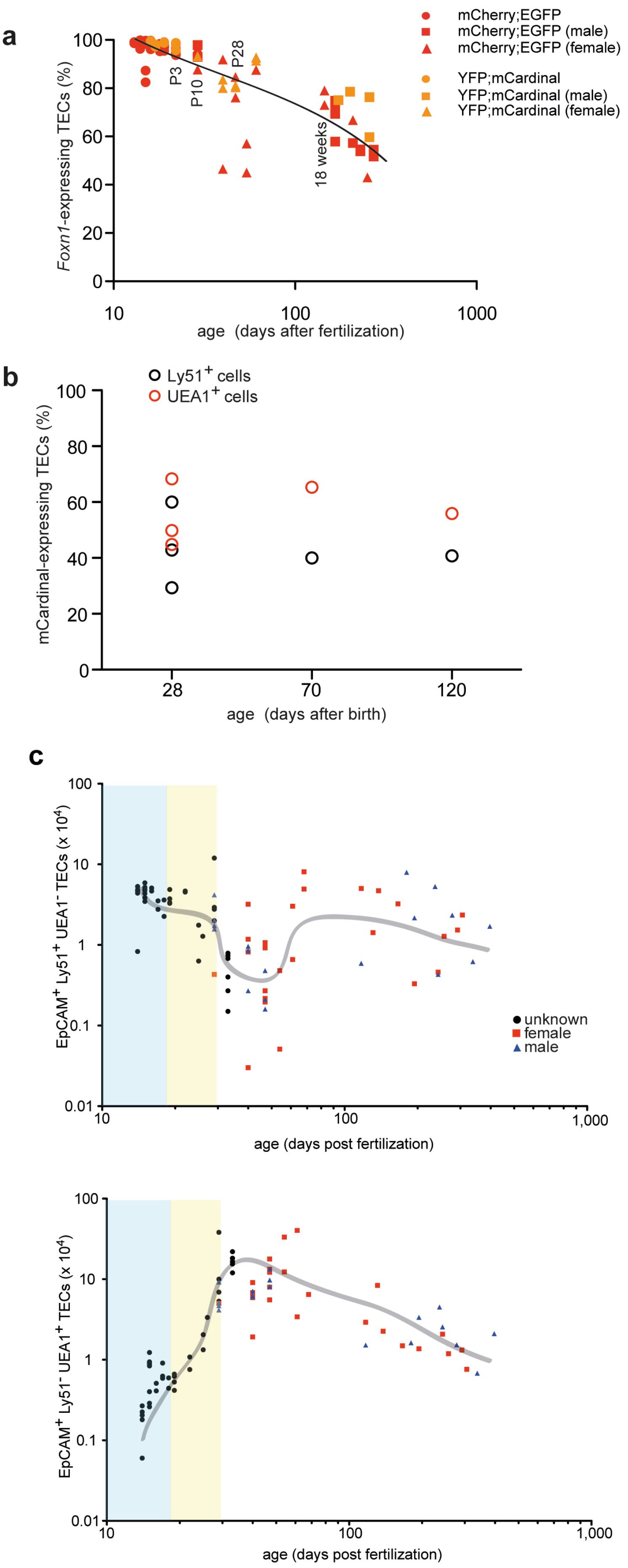
a
Foxn1-expressing TECs (%)
100
80
60
40
20
0
10
100
1000
age (days after fertilization)
P3
P10
P28
18 weeks
mCherry;EGFP
mCherry;EGFP (male)
mCherry;EGFP (female)
YFP;mCardinal
YFP;mCardinal (male)
YFP;mCardinal (female)
b
Ly51+ cells
UEA1+ cells
mCardinal-expressing TECs (%)
28
70
120
age (days after birth)
c
EpCAM+ Ly51+ UEA1- TECs (x 10^4)
100
10
1
0.1
0.01
1,000
age (days post fertilization)
unknown
female
male
EpCAM+ Ly51- UEA1+ TECs (x 10^4)

Supplementary Figure 2. Characteristics of thymic epithelial cells. (**a**) Fraction of TECs expressing *Foxn1* as a function of age, as determined by transgene fluorescence in the indicated genotypes. Note that TECs of males and females do not appreciably differ with respect to the fraction of *Foxn1*-negative cells. (**b**) Both $Ly51^{+}$ and $UEA1^{+}$ cells lose *Foxn1* expression. TECs were isolated from the thymi of *Foxn1:YFP*^{NLS}*;Foxn1:mCardinal*; *Foxn1:Cre*;*RosaR26SLSYFP* quadruple transgenic mice to record both past and present *Foxn1* expression (see text for details). (**c**) Absolute numbers of $Ly51^{+}UEA1^{-}$ cells (**c**) and $Ly51^{-}UEA1^{+}$ cells (**d**) among $EpCAM^{+}$ TECs as determined by flow cytometry (see Fig. 2).

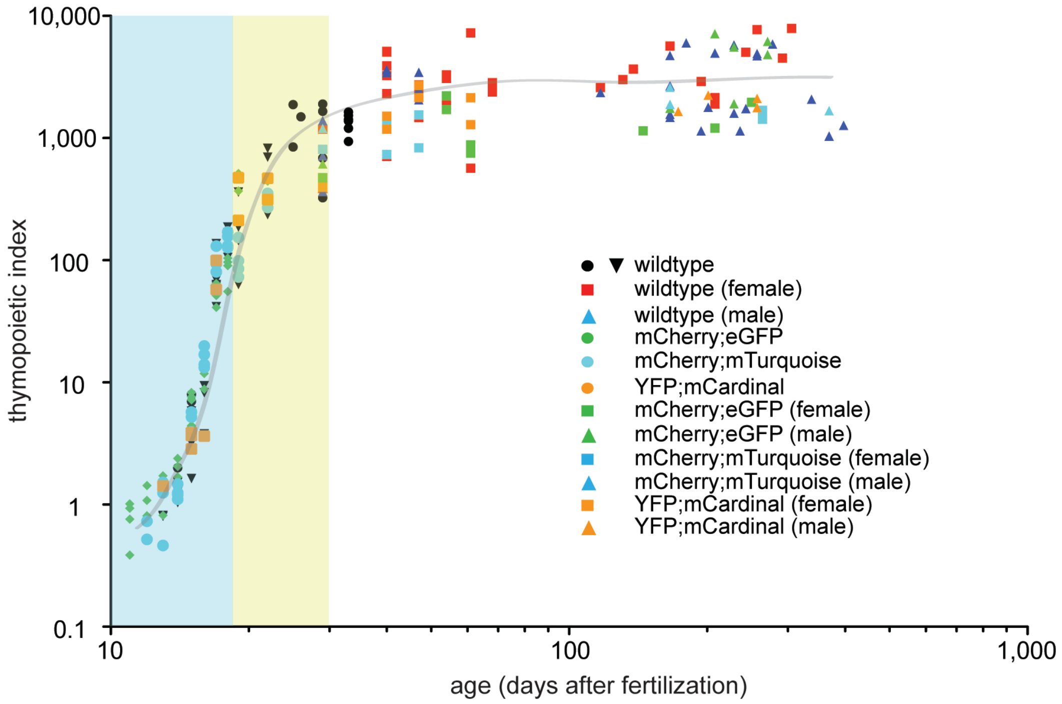

Supplementary Figure 3. Age-dependent changes of the thymopoietic index, as determined by flow cytometry following enzymatic tissue dissociation. The thymopoietic index is expressed as the ratio of $CD45^+$ and $EpCAM^+$ cells. Each symbol represents a single animal with genotypes and sex (where known) indicated in the figure key; the two wild-type symbols represent mice of two cohorts analysed in different years. The blue shading denotes the embryonic phase, the yellow shading marks the perinatal period until P10.

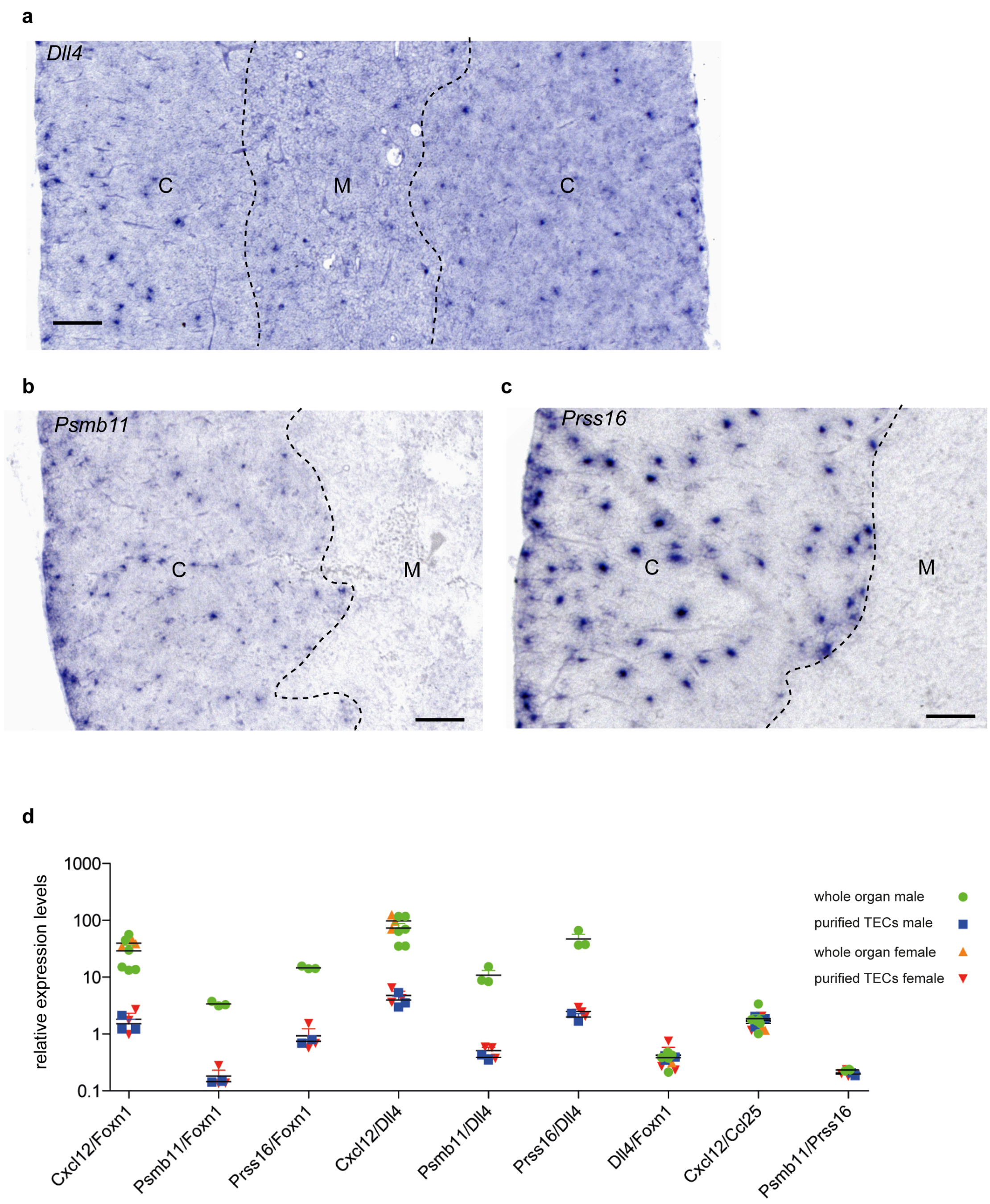

Supplementary Figure 4. Expression signature of TEC-specific genes. RNA *in situ* hybridization to localize TECs expressing *Dll4* (**a**), *Psmb11* (**b**), and *Prss16* (**c**) in sections of a 4-week-old mouse. The positions of cortex (C) and medulla (M) are indicated. Scale bars, 0.1mm. (**d**) Expression levels of epithelial-specific genes with a regio-specific signature in the thymus using RNA extracted from purified TECs or whole thymi. Each symbol represents a single mouse. The fold changes between whole organ and purified TECs are indicated.

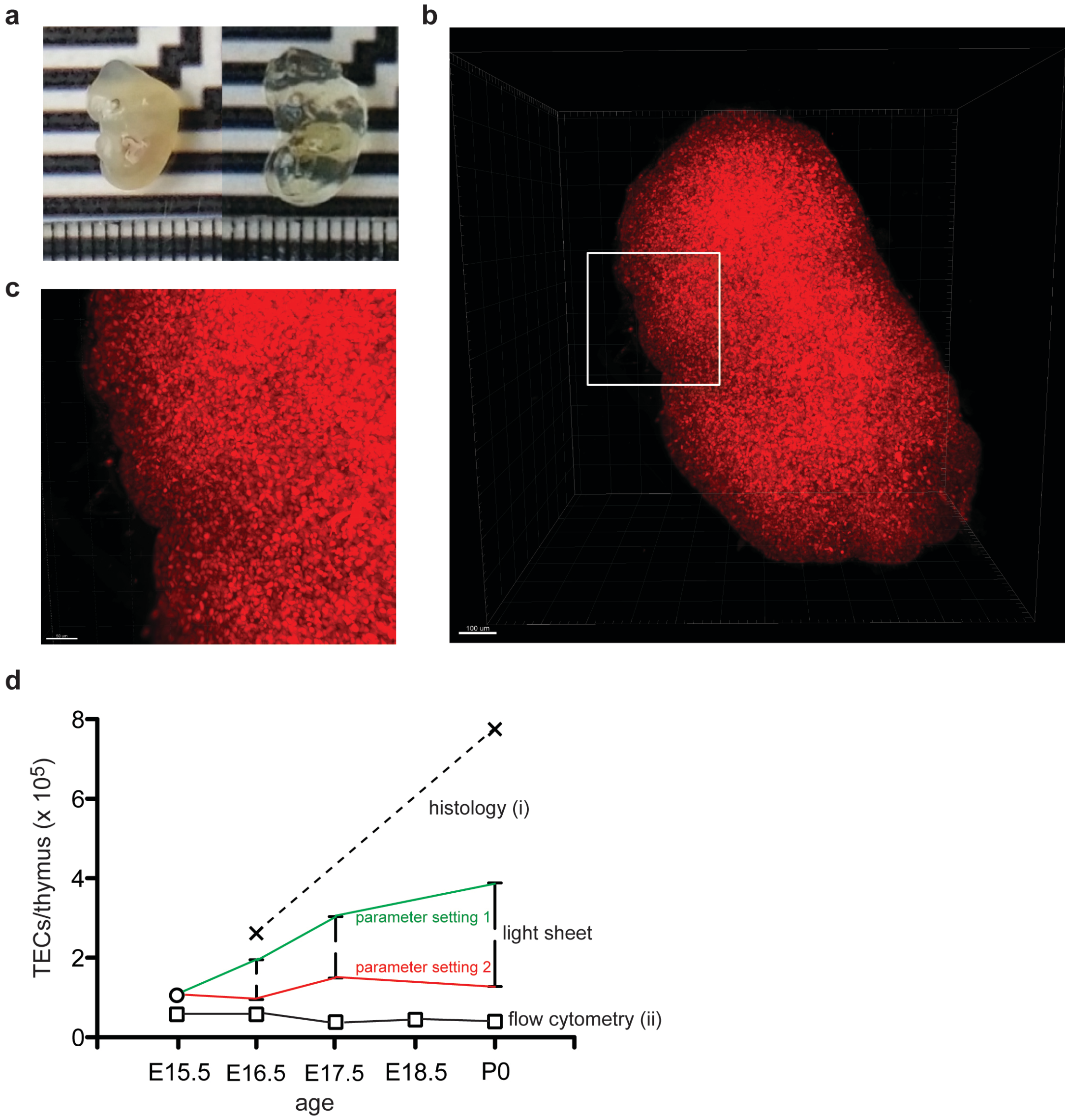

Supplementary Figure 5. Determination of the absolute number of TECs. (**a**) Demonstration of the effectiveness of tissue clearing of E12.5 embryos. The left embryos was fixed with further treatment, whereas the right embryo was additionally subjected to clearing after fixation. (**b**) Reconstructed image indicating the distribution of TEC nuclei of *Foxn1:mCherry*^{NLS};*Foxn1:Turquoise*^{mem} transgenic mice in the intact organ as determined by light-sheet microscopy. Scale bar, 0.1 mm. (**c**) Higher magnification of a selected region of (b). Scale bar, 0.05 mm. (**d**) Summary of three different methods to determine the absolute number of TECs. Data are taken (i) for the histological procedure from Figure 3c,d; (ii) for flow cytometric measurements are taken from Figure 2b. The values presented for the four time points of the light-sheet determinations are from two different parameters for nucleus size; 6 mm (green line), 10 mm (red line). For details, see text.

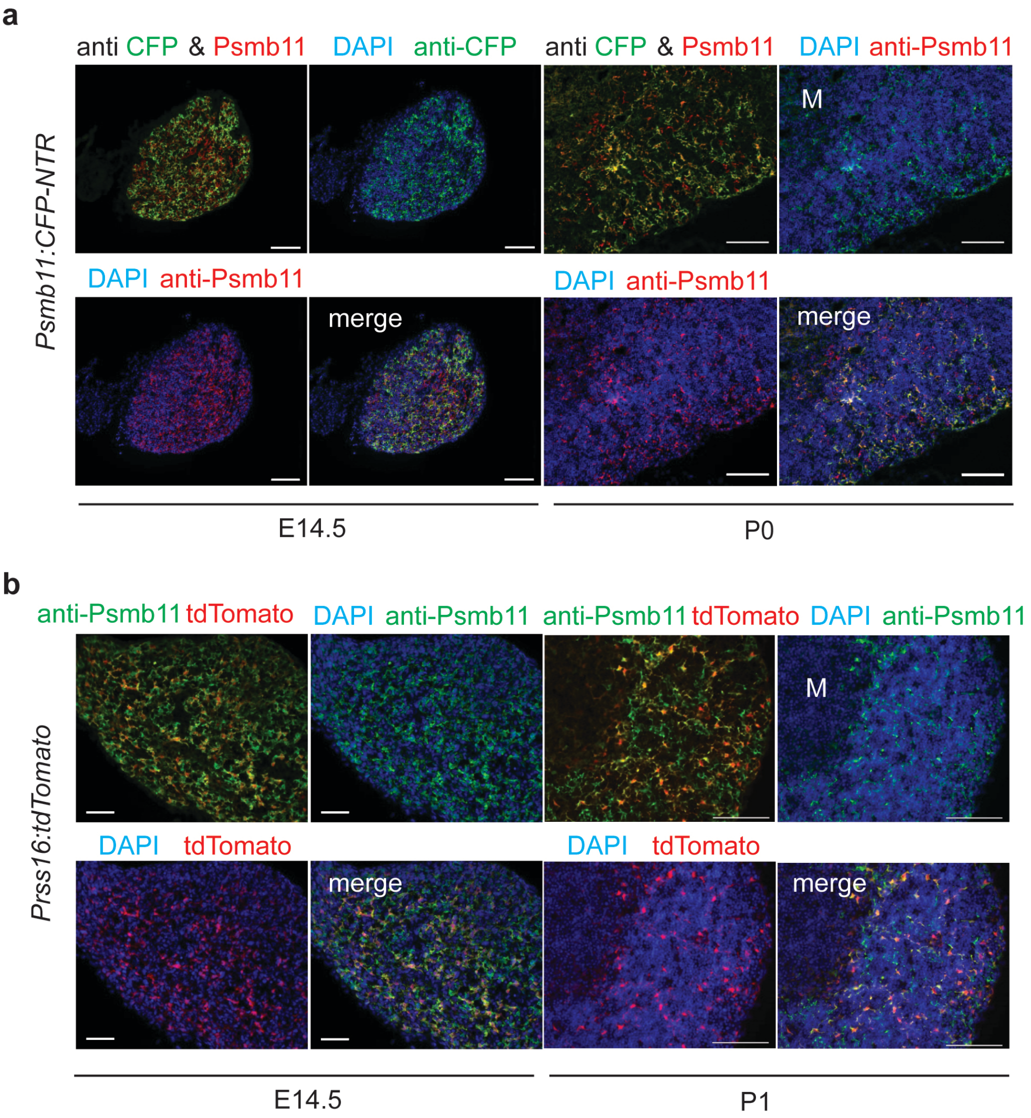

Supplementary Figure 6. Characterization of cTEC-specific transgenic lines. (**a**) The activity of the *Psmb11* promoter is restricted to thymic epithelial cells. The anti-CFP staining identifies expression of the transgene, the anti-Psmb11 antibody marks epithelial progenitor and cTECs. Note that most TECs in the cortex are double-positive, indicating that the transgenic construct largely reflects the endogenous *Psmb11* expression pattern. (**b**) The activity of the *Prss16* promoter is restricted to a subset of cortical thymic epithelial cells. The red fluorescence identifies expression of the transgene, the anti-Psmb11 antibody marks epithelial progenitor and cTECs. Note that a large fraction of transgene-expressing cells is also positive for Psmb11 (yellow); some cTECs are positive for either the transgene (red) or Psmb11 (green). Scale bars, 0.1mm.

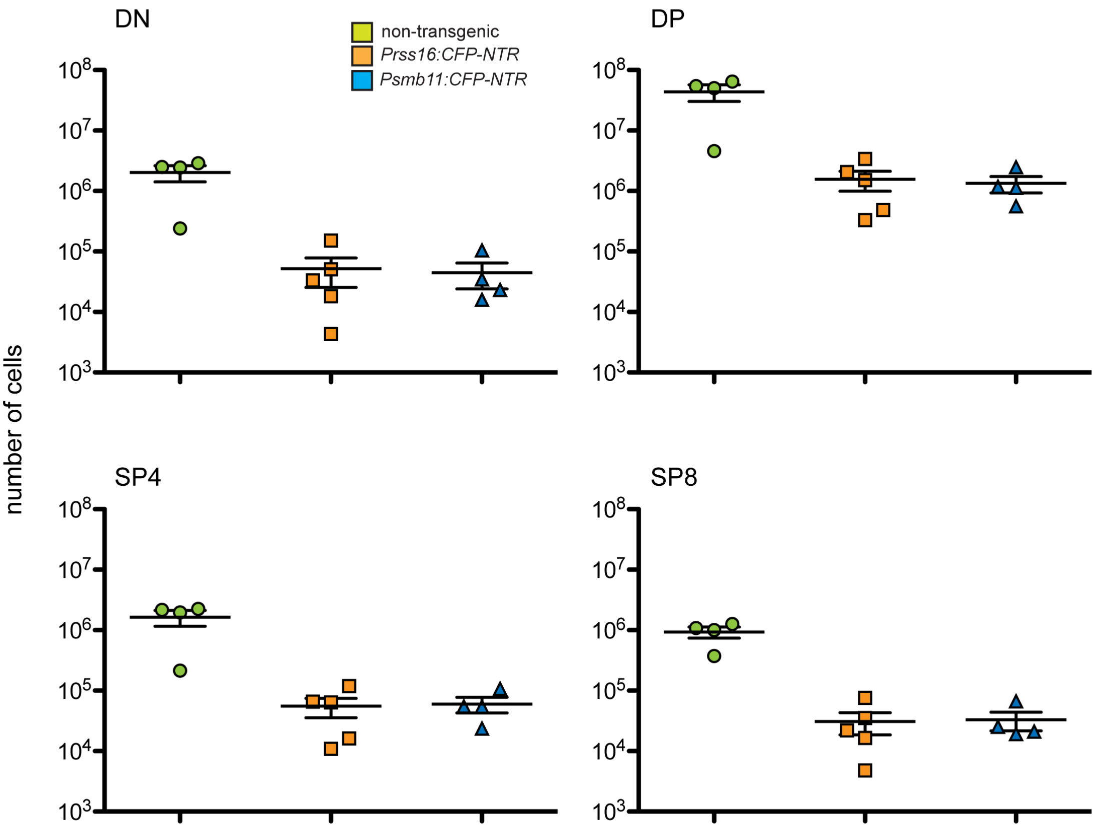

Supplementary Figure 7. Thymocyte subsets of regenerating thymi after subtotal ablation of cTEC-like cells. For experimental strategy, see Fig. 5. Total number of TECs (left panel) and $CD45^+$ haematopoietic cells as determined by flow cytometry following enzymatic dissociation at P7; each symbol represents a single mouse. Although the absolute numbers of cells in all subsets are significantly lower in regenerating thymi as compared to control (at least $P<0.04$), the relative ratios of subsets are not significantly different from each other in all three conditions with the exception of a significant elevation ($P=0.03$) of the percentage of CD4 single-positive cells in *Psmb11:CFP-NTR* transgenics relative to the *Prss16:CFP-NTR* transgenics.
